# Solvent-Dependent Reactivity of Fe(CO)_5_ under Superacidic and Oxidative Conditions

**DOI:** 10.1021/jacs.4c09595

**Published:** 2025-01-14

**Authors:** Willi R. Berg, Marc Reimann, Robin Sievers, Susanne M. Rupf, Johanna Schlögl, Kilian Weisser, Konstantin B. Krause, Christian Limberg, Martin Kaupp, Moritz Malischewski

**Affiliations:** † Institut für Anorganische Chemie, Freie Universität Berlin, Fabeckstraße 34–36, D-14195 Berlin, Germany; ‡ Institut für Chemie, Technische Universität Berlin, Straße des 17. Juni 135, 10623 Berlin, Germany; § Institut für Chemie, Humboldt-Universität zu Berlin, Brook-Taylor-Straße 2, 12489 Berlin, Germany

## Abstract

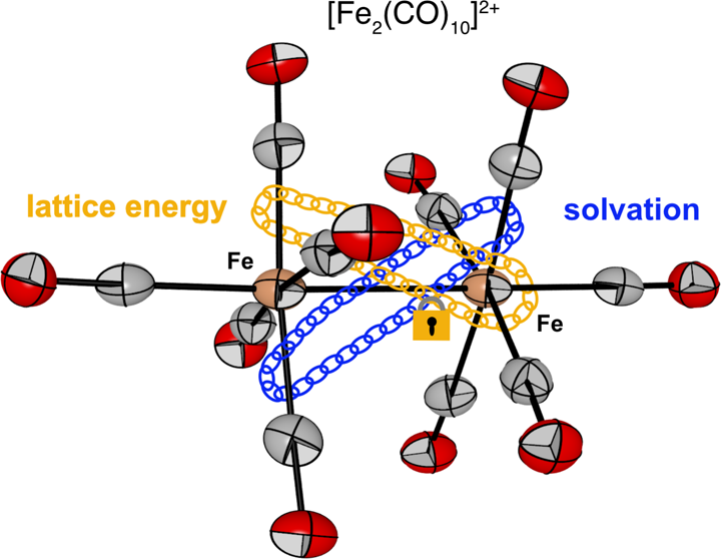

Herein, we report the solvent-dependent reactivity of Fe(CO)_5_ toward AsF_5_ in either anhydrous HF or liquid SO_2_. The reaction of Fe(CO)_5_ with the superacid HF/AsF_5_ leads to the protonation of the iron center and allows for the first-time structural characterization of [FeH(CO)_5_]^+^ in the solid state, representing one of the most acidic transition metal hydride complexes to ever be isolated and structurally characterized. In the aprotic but oxidation-stable solvent SO_2_, Fe(CO)_5_ is oxidized and dimerized to [Fe_2_(CO)_10_]^2+^, which is isoelectronic with well-known Mn_2_(CO)_10_. [Fe_2_(CO)_10_]^2+^ is the first structurally characterized example of a homoleptic dinuclear transition metal carbonyl cation. Together with Fe(CO)_5_ and [Fe(CO)_5_]^+•^, it is a rare example of an iron-centered triad from which the neutral, the radical cationic, and the dimerized dicationic species have been structurally and spectroscopically characterized. All characterizations are well supported by quantum chemical calculations. We also make the argument that the dimerization of [Fe(CO)_5_]^+•^ is largely dependent on the employed solvent.

## Introduction

Iron carbonyl complexes are ubiquitous in organotransition metal chemistry and exist in a great structural variety (with or without auxiliary ligands) with formal oxidation states ranging from −II in [Fe(CO)_4_]^2–^ up to +IV in [Cp*_2_Fe(CO)]^2+^ (Cp* = η^5^-C_5_Me_5_).^1,2^ The most important homoleptic iron carbonyl, Fe(CO)_5_, was discovered shortly after Ni(CO)_4_ by Mond.^3^

**Figure 1:**
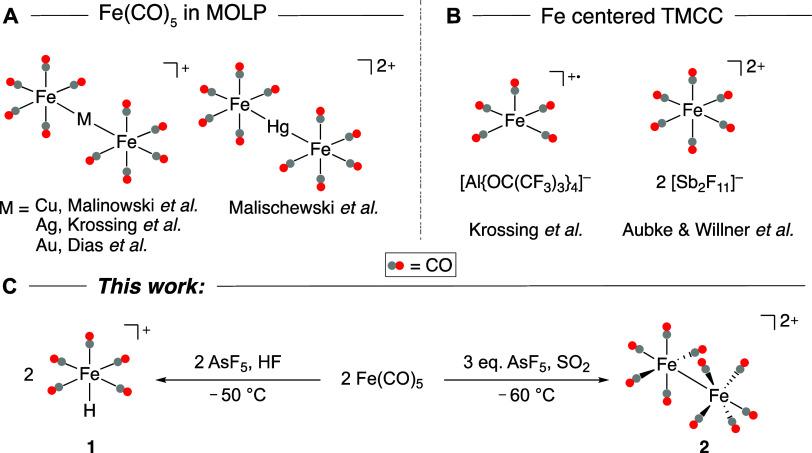
(A) Fe(CO)_5_ as a ligand in MOLP^7,9,10^ and (B) homoleptic Fe-centered TMCCs characterized in the solid state.^13,14^ (C) The reactivity of Fe(CO)_5_ with AsF_5_ in either HF or SO_2_ resulting in protonation or oxidation, respectively.

Despite its metal in a low oxidation state, Fe(CO)_5_ is a weak base and protonation is only achieved under superacidic conditions.^4^ Mass spectrometry,^5^^57^Fe Mössbauer spectroscopy,^6^ and vibrational spectroscopy^4^ indicate an iron bound hydride. However, [FeH(CO)_5_]^+^ was never structurally characterized due to its high reactivity and instability. Besides its Brønsted basicity, Fe(CO)_5_ can act as a Lewis base toward (di)cationic Lewis acidic metal centers.^6−10^ In recent years, there has been increasing interest in Lewis base–Lewis acid metal adducts, specifically metal-only Lewis pairs (MOLP).^11^ While Braunschweig et al. coordinated Fe(CO)_5_ to the main group Lewis acid GaCl_3_,^12^ Fe(CO)_5_ has also been successfully coordinated to the complete triad of group 11 coinage metal cations Cu^+^/Ag^+^/Au^+^ (Figure 1A).^7,9^ Quantum chemical calculations on [M{Fe(CO)_5_}_2_]^+^ (M = Cu, Ag, Au) indicate that the [Fe(CO)_5_] → M^+^ ← [Fe(CO)_5_] donation is significantly stronger than the [Fe(CO)_5_] ← M^+^ → [Fe(CO)_5_] backdonation.^9^

A different bonding situation is assigned for the isoelectronic [Hg{Fe(CO)_5_}_2_]^2+^ adduct (Figure 1A) in which the dominant dative bonds come from the σ-donation of Hg(0) into the vacant orbitals of a formal [Fe_2_(CO)_10_]^2+^ acceptor fragment.^10^

In this context, the oxidizability of Fe(CO)_5_ is of general interest. Whereas oxidation under CO atmosphere using strong oxidants (XeF_2_ in HF-SbF_5_) gives [Fe(CO)_6_]^2+^,^13^ the 17 VE species [Fe(CO)_5_]^+•^ (Figure 1B) is accessible by using strong one-electron oxidizers with the weakly coordinating [Al{OC(CF_3_)_3_}_4_]^−^ aluminate anion in organic solvents.^14^ This 17 VE transition metal carbonyl cation (TMCC) was fully characterized spectroscopically and structurally but was not found to dimerize, unlike its isoelectronic analogue [Mn(CO)_5_]^•^. Seventeen VE monomeric fragments (e.g., [Mn(CO)_5_]^•^, [Re(CO)_5_]^•^, [Co(CO)_4_]^•^) are usually observed to dimerize as shown for Mn_2_(CO)_10_, Re_2_(CO)_10_, and Co_2_(CO)_8_.^15−17^

Additionally, oligonuclear homoleptic TMCCs with M–M bonds are still scarce, with [M_3_(CO)_14_]^2+^ (M = Ru, Os) and [Ru_2_(CO)_10_]^2+^ being the only complexes structurally characterized by single-crystal X-ray diffraction (scXRD).^18,19^ Besides mass spectrometric investigations of clustered TMCCs,^20^ only [Hg_2_(CO)_2_]^2+^ and [Pt_2_(CO)_6_]^2+^ have been reported in the condensed phase.^21^ However, the instability of these complexes did not allow for characterization by scXRD.

In previous studies, pnictogen pentafluorides EF_5_ (E = As, Sb) in liquid SO_2_ functioned as powerful oxidizers, allowing for the isolation of organometallic dications.^22^ When used in anhydrous HF (aHF), EF_5_ (E = P, As, Sb) form superacids, e.g., aHF/PF_5_, generating reactive metal hydride species through protonation (e.g., isolation of protonated ferrocene).^23^ Consequently, in this study, we wondered whether [FeH(CO)_5_]^+^ or [Fe_2_(CO)_10_]^2+^ (isoelectronic to Mn_2_(CO)_10_) could be obtained by reacting Fe(CO)_5_ with EF_5_ in either aHF or SO_2_.

## Results and Discussion

**Figure 2:**
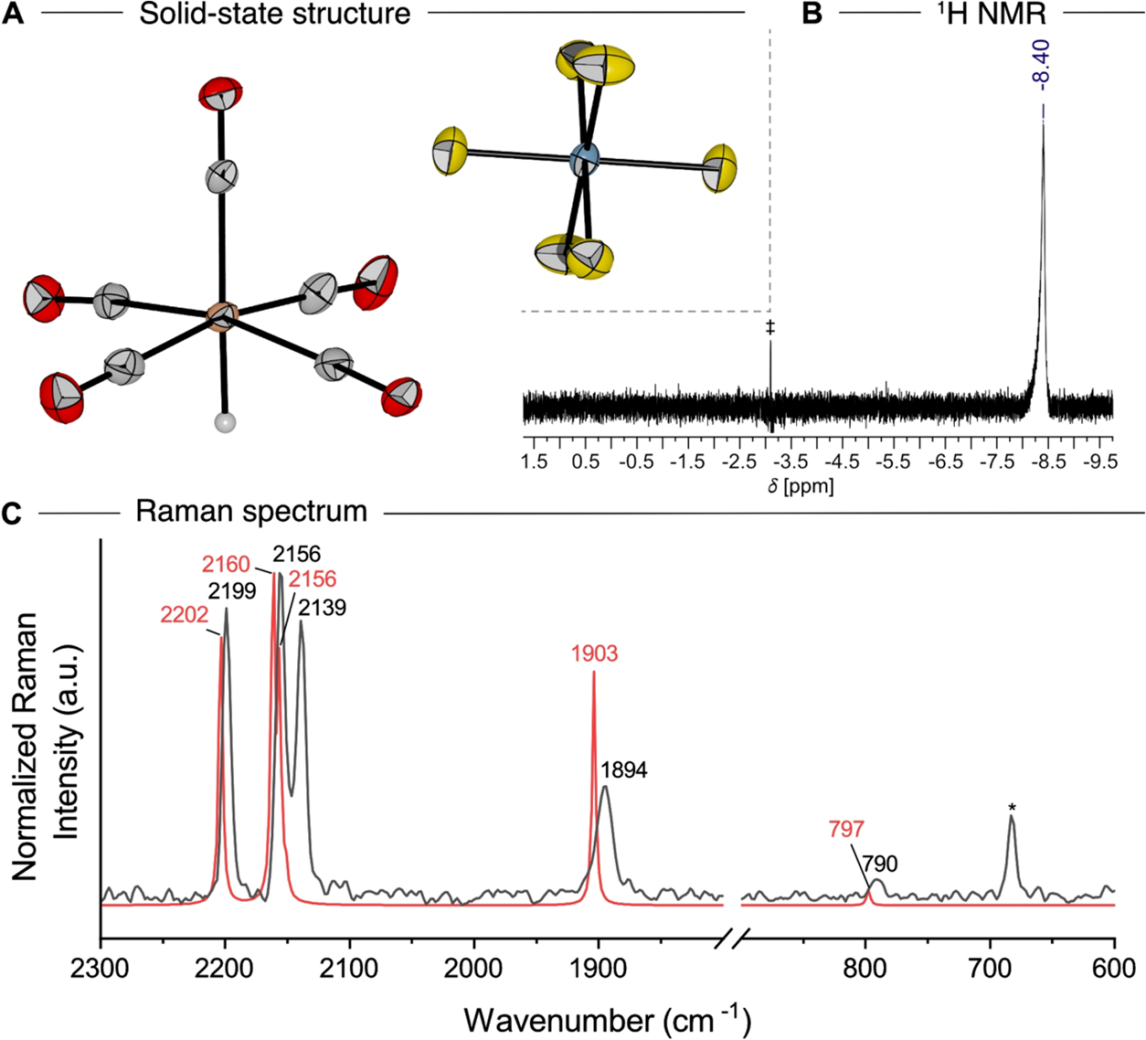
(A) Molecular structure in the solid state of [FeH(CO)_5_]^+^ and [AsF_6_]^−^. Displacement ellipsoids are shown at a probability of 50%. Color code: orange = iron, gray = carbon, red = oxygen, light gray = hydrogen, blue = arsenic, yellow = fluorine. (B) The ^1^H NMR spectrum of [FeH(CO)_5_]^+^ [AsF_6_]^−^ in aHF. ^‡^ denotes an instrument artifact due to the centering of the measured spectrum at δ = −3 ppm and is therefore no real signal. (C) Experimental (black) and calculated (red, B3LYP-D3(BJ)/def2-TZVPP) Raman spectrum of [FeH(CO)_5_]^+^ [AsF_6_]^−^ in the regions 2300–1800 and 900–700 cm^–1^. Calculated frequencies are scaled by 0.968 according to Duncan et al.^25^ Asterisk corresponds to anion bands.

Indeed, reacting Fe(CO)_5_ with AsF_5_ in aHF results in the desired protonated species **1** (Figure 1C). Slowly cooling a solution of [FeH(CO)_5_]^+^ [AsF_6_]^−^ in aHF from room temperature (rt) to −70 °C gave single crystals suitable for scXRD, providing the first structurally characterized [FeH(CO)_5_]^+^ moiety (Figure 2A). [FeH(CO)_5_]^+^ [AsF_6_]^−^ crystallizes in monoclinic space group *C*2/*c*. The structure of [FeH(CO)_5_]^+^ displays a square pyramidal arrangement of the CO ligands and is structurally similar to the literature-known radical cation [Fe(CO)_5_]^+•^. However, [FeH(CO)_5_]^+^ features an additional hydrido ligand, which results in an overall pseudo-octahedral geometry. In both complexes, the iron center is shifted out of the equatorial plane toward the axial CO ligand. The angles spanned by opposing equatorial CO ligands in [FeH(CO)_5_]^+^ [AsF_6_]^−^ are 166.6(2) and 168.5(2)°, similar to the ones observed in [Fe(CO)_5_]^+•^ [Al{OC(CF_3_)_3_}_4_]^−^ (166.0(2) and 169.1(2)°), resulting in C_2v_ symmetry in contrast to the theoretically expected C_4v_ symmetry, most likely due to additional interactions in the solid state.^14^ The observed Fe–H bond length of 142.0(5) pm is shorter than the calculated ones (B3LYP-D3(BJ)/def2-TZVPP 150.6 pm, r^2^SCAN-3c^24^ 150.7 pm). The remaining differences may be attributed to stabilization by counterions and general dielectric effects in the solid state. It should also be noted, however, that the hydride ligand is difficult to localize experimentally next to the heavy iron center. The equatorial Fe–C bond lengths average to 184.8(3) pm, and all individual equatorial Fe–C bond lengths are within ≤3σ. The axial Fe–C bond length is 188.7(3) pm, and the average C–O bond length is 111.8(4) pm.

The measured IR and Raman spectra are in good agreement with the spectra calculated at the B3LYP-D3(BJ)/def2-TZVPP level (Figure 2C). The characteristic Fe–H stretching frequency is observed in the Raman spectrum (Figure 2C) at ν̃ = 1894 cm^–1^ and agrees well with the calculated spectrum. A Fe–H wagging mode is observed in both the IR and Raman spectra at ν̃ = 784 and 790 cm^–1^, respectively. The nondegeneracy of the Fe–H wagging mode is a result of the lower C_2v_ symmetry of **1** in the solid state. Additionally, CO stretching frequencies are observed at ν̃ = 2199, 2156, and 2139 (Raman, Figure 2C) and 2124 and 2095 (IR, Figure S2) cm^–1^.

The ^1^H NMR spectrum shows a typical metal hydride shift at δ = −8.4 ppm (Figure 2B). In the ^13^C NMR spectrum, two signals with an intensity ratio of 5:1 were observed at 192.0 and 190.3 ppm (Figure S6). This indicates a general high-field shift in comparison to Fe(CO)_5_ (216.0 and 208.1 ppm) or isoelectronic [MnH(CO)_5_] (211.4 and 210.8 ppm), which is in agreement with the literature.^26^ The ^19^F NMR shows a broad saddle-like signal at δ = −70.2 ppm corresponding to the [AsF_6_]^−^ counterion^27^ (Figure S5), as well as a highly intense signal for HF, which was used as the solvent.

Since a study from 1976 had reported the ^57^Fe Mössbauer shifts of [FeH(CO)_5_]^+^ [PF_6_]^−^ prepared by reaction between Fe(CO)_5_ and PF_5_ in anhydrous HCl as a solvent,^6^ we were curious to see if the spectroscopic data matched the ones from our samples. The zero-field ^57^Fe Mössbauer spectrum (solid sample measured at 14 K, Figure S7) displays a single symmetric doublet with an isomer shift of δ = −0.08 mm s^–1^ and a quadrupole splitting (QS) of Δ*E* = 1.40 mm s^–1^, which is in good agreement with the spectrum of [FeH(CO)_5_]^+^ [PF_6_]^−^ reported in the literature.^6^ In contrast to [FeH(CO)_5_]^+^ [PF_6_]^−^, which rapidly decomposes at rt,^4^ [FeH(CO)_5_]^+^ [AsF_6_]^−^ can be handled at room temperature and shows no decomposition stored over several days at −36 °C under inert conditions.

To the best of our knowledge, [FeH(CO)_5_]^+^ [AsF_6_]^−^ is one of the most acidic metal hydride species to have ever been structurally characterized by scXRD.^28^ In order to rank the acidity of such complexes, the calculation of proton affinities of the corresponding neutral species/Brønsted bases in the gas phase is particularly useful because it also allows comparability with any other molecule of the same charge. DFT calculations (B3LYP-D3(BJ)/def2-TZVPP) reveal a proton affinity of Fe(CO)_5_ of 815 kJ/mol, which is in between weak organic bases as tetrahydrofuran (832 kJ/mol) and acetonitrile (789 kJ/mol). Since many metal hydrides are only weak acids, presumably, the most acidic complex so far reported is actually a dihydrogen complex ([Re(CO)_5_H_2_]^+^; no characterization by scXRD).^29^ However, the calculated proton affinity of [Re(CO)_5_H] (835 kJ/mol) indicates that it is approximately 20 kJ mol^–1^ more basic than that of Fe(CO)_5_ (Table S7).

While AsF_5_ in HF typically leads to protonation, it is far more oxidizing in liquid SO_2_. Consequently, Fe(CO)_5_ is oxidized by AsF_5_ in SO_2_ resulting in [Fe_2_(CO)_10_]^2+^ [AsF_6_]^–^_2_ (Figure 1C) as a yellow solid. The volatile byproduct AsF_3_ is easily removed under vacuum.

**Figure 3:**
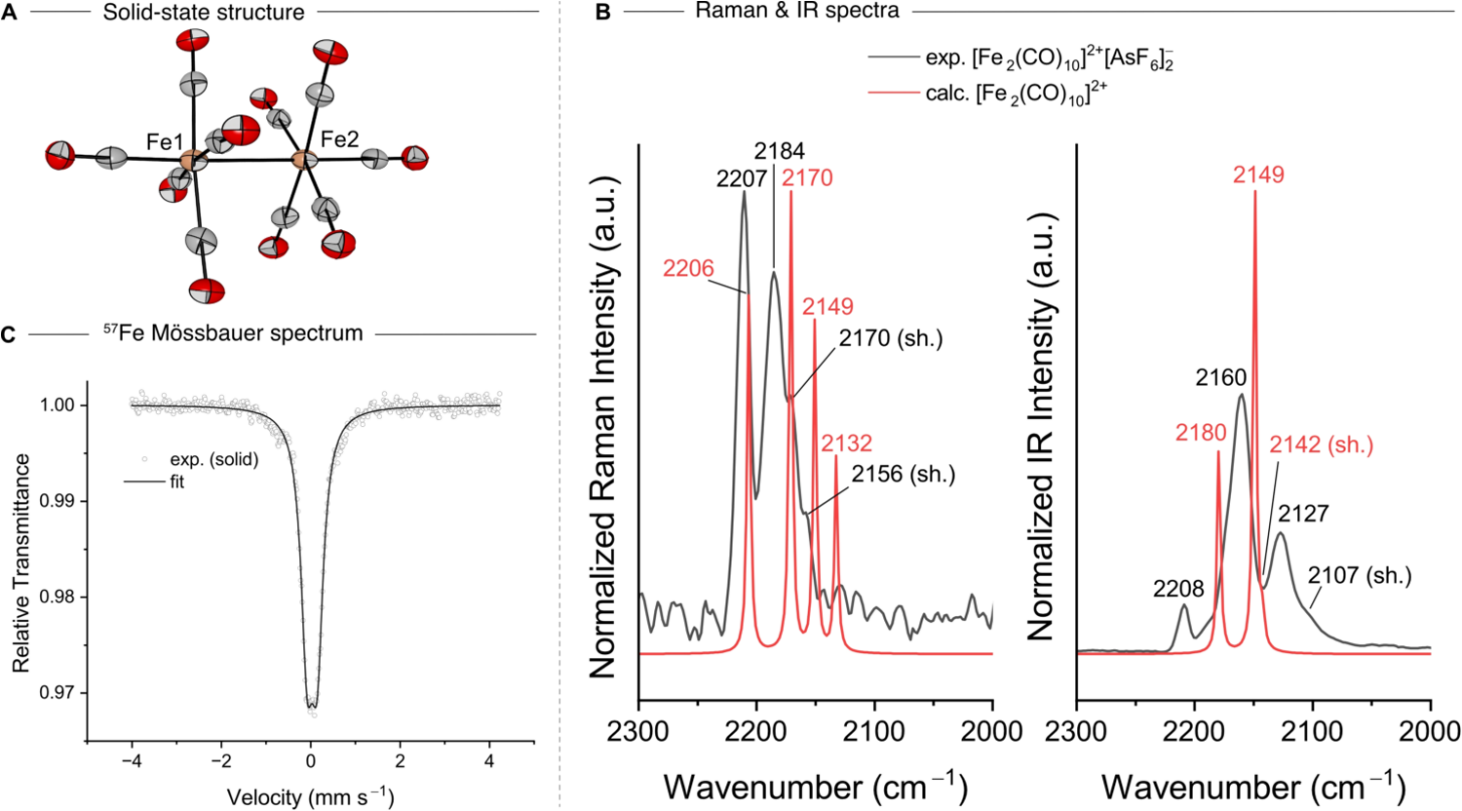
(A) Molecular structure in the solid state of the dimeric dication in [Fe_2_(CO)_10_]^2+^_2_ [FeH(CO)_5_]^+^ [AsF_6_]^–^_5_. Other molecules have been omitted for the sake of clarity. Displacement ellipsoids are shown at a probability of 50%. Color code: orange = iron, gray = carbon, red = oxygen. Selected bond length [pm]: Fe–Fe 282.4(2) pm. (B) Raman (left) and IR (right) spectra in the CO region of 2300–2000 cm^–1^, comparing experimental (black) spectra of [Fe_2_(CO)_10_]^2+^ [AsF_6_]^–^_2_ with the calculated (red, B3LYP-D3(BJ)/def2-TZVPP) spectra of [Fe_2_(CO)_10_]^2+^. Calculated frequencies are scaled by 0.968 according to Duncan et al.^25^ (C) The zero-field ^57^Fe Mössbauer spectrum of solid [Fe_2_(CO)_10_]^2+^ [AsF_6_]^–^_2_ measured at 14 K. The solid black line represents the numerical fit of the measured experimental data indicated by the gray circles; a slight broadening of the signal base may be attributed to the presence of a second species (6%, see Figure S14, presumably [FeH(CO)_5_]^+^[AsF_6_]^−^).

Single crystals of [Fe_2_(CO)_10_]^2+^[AsF_6_]^–^_2_·2SO_2_ of moderate quality were obtained upon cooling from rt to −70 °C (Figure S15). The compound crystallizes in orthorhombic space group *P*2_1_2_1_2_1_. The solid-state structure of [Fe_2_(CO)_10_]^2+^ reveals a pseudo-octahedral coordination structure on both iron centers with a staggered conformation of the eight equatorial CO ligands (Figure 3A). Additionally, the eight equatorial CO ligands are slightly bent toward the second metal center, resulting in Fe–Fe–C angles of 85.6(5)–89.7(6)°. The Fe–Fe bond length of 282.7(3) pm is shorter than the Mn–Mn bond in its isoelectronic counterpart Mn_2_(CO)_10_ (*d*_Mn–Mn_ = 290.3(2) pm),^30^ despite greater electrostatic repulsion of the cationic [Fe(CO)_5_]^+•^ fragments. In serendipitously obtained crystals of [Fe_2_(CO)_10_]^2+^_2_ [FeH(CO)_5_]^+^ [AsF_6_]^–^_5_ (Figure S16), the Fe–Fe bond distance is 282.4(2) pm. The shorter bond can be explained by the charge-induced contraction of the bonding orbital in the [Fe(CO)_5_]^+^ fragments relative to the [Mn(CO)_5_] units, as can be seen from the reduction of the Pauli-repulsion contribution to the bond (Δ*E*_Pauli_, Table 1) in energy decomposition analysis (EDA). This also most likely increases the charge-shift bond (CSB)^31^ character of the Fe–Fe bond compared to the Mn–Mn bond in Mn_2_(CO)_10_, which is a well-established example of a CSB.^32^ It should, however, be noted that the Fe–Fe bond is metastable in the gas phase. This is due to the Coulomb repulsion (“Coulomb explosion”) of the charged fragments, which leads to a sign change in the electrostatic contribution to the bond (Δ*E*_Elstat._) when going from the Mn to the Fe compound (Table 1). In solution, the bond is stabilized by a dielectric interaction with the solvent (Δ*E*_COSMO_). The situation is reminiscent of the dimerization of other monomeric charged fragments to more highly charged aggregates, which are also typically only stabilized by the interactions with the anions in the solid state.^33^

**Table 1:** Results of Energy Decomposition Analysis and the Eigenvalue of the Most Important Contribution in an ETS-NOCV Analysis (Δ*E*_NOCV_) of the M–M Bond in Mn_2_(CO)_10_ and [Fe_2_(CO)_10_]^2+^ (Referring to a Homolytic Bond Dissociation into Two Unrelaxed Doublet Fragments) at the BP86/TZP//r^2^SCAN-3c Level of Theory in kJ/mol

	ε^a^	Δ*E*_Pauli_	Δ*E*_Elstat._	Δ*E*_Orb.Int._	Δ*E*_COSMO_^b^	Δ*E*_Int_	Δ*E*_NOCV_
Mn–Mn		257.5	–166.5	–217.5		–195.1	–189.5
Mn–Mn	13.0	250.3	–152.9	–225.6	6.6	–190.2	–190.6
Mn–Mn	20.0	249.9	–152.1	–226.1	7.1	–190.0	–190.7
Fe–Fe		158.0	266.1	–226.6		135.6	–177.4
Fe–Fe	13.0	151.8	310.7	–270.1	–210.6	–80.1	–183.1
Fe–Fe	20.0	151.5	312.7	–272.2	–216.3	–86.2	–183.5
Fe–Fe	23.0	151.4	313.2	–272.7	–217.7	–87.7	–183.6
Fe–Fe		186.7	252.5	–242.4		133.7	–189.2
Fe–Fe^c^	13.0	179.3	299.5	–286.9	–212.0	–83.2	–195.4
Fe–Fe^c^	23.0	178.8	302.0	–289.5	–219.1	–90.9	–195.8

a Dielectric constant used in COSMO calculations.

b Contribution only includes the electrostatic interaction energy with the cavity charges. No cavity formation contributions were considered.

c Obtained at a relaxed structure with a fixed Fe–Fe bond length of 282 pm.

Interestingly, our calculations provide a significantly larger bond length (r^2^SCAN-3c = 289.1 pm and B3LYP-D3(BJ)/def2-TZVPP = 297.2 pm). A relaxed scan along the Fe–Fe bond provided a very shallow potential energy surface that changes by only 4 kJ/mol between 275 and 295 pm (Table S6). The shorter experimental bond length is very likely due to intermolecular interactions.

The structure of the observed vibrational bands for [Fe_2_(CO)_10_]^2+^ agrees with the calculated (B3LYP-D3(BJ)/def2-TZVPP) frequencies in both the IR and Raman spectra (Figure 3B), with characteristic CO bands in the IR spectrum at ν̃ = 2208, 2160, 2127, and 2107 cm^–1^ and in the Raman spectrum at ν̃ = 2207, 2184, 2170, and 2156 cm^–1^. The appearance of a band at 2208 cm^–1^ in the IR spectrum is probably caused by solid-state interactions with the [AsF_6_]^−^ counteranion, possibly causing distortion of the coordination arrangement. [Fe_2_(CO)_10_]^2+^ can also be classified as a nonclassical carbonyl complex, considering that the averaged value of ν̃(CO) is above the threshold of free CO of ν̃ = 2143 cm^–1^.^34^

Although a clear blueshift of the CO bands in comparison to Fe(CO)_5_ (IR: ν̃ = 2020, 1989 cm^–1^, Raman: ν̃ = 2114, 2028, 1985 cm^–1^)^35^ and [Fe(CO)_5_]^+•^ (IR: ν̃ = 2128, 2113, 2082 cm^–1^)^14^ is apparent, the values are not as extreme as in [Fe(CO)_6_]^2+^ (IR: ν̃ = 2204 cm^–1^; Raman: ν̃ = 2241, 2220 cm^–1^).^13^

[Fe_2_(CO)_10_]^2+^ was further investigated by zero-field ^57^Fe Mössbauer spectroscopy, displaying one major signal with an isomer shift of δ = 0.03 mm s^–1^, which is in agreement with the expectations for an Fe(I) center. However, the dication exhibits an astonishingly small QS of Δ*E* = 0.23 mm s^–1^ (Figure 3C), usually observed for Fe centers, which have an almost spherically symmetric environment (e.g., the ^57^Fe Mössbauer spectrum of the homoleptic octahedral [Fe(CO)_6_]^2+^^13^ with the entire t_2g_ orbital set filled, features a single line experiencing negligible QS). However, neither the electron distribution nor the ligation is symmetric in the case of [Fe_2_(CO)_10_]^2+^. Therefore, the ^57^Fe Mössbauer parameters of [Fe(CO)_5_]^+•^ and [Fe_2_(CO)_10_]^2+^ have been calculated using structures optimized at the r^2^SCAN-3c level and the protocol proposed in ref (36) (Table 2). As we have observed a very flat potential energy surface along the Fe–Fe bond, we obtained Mössbauer parameters for various bond lengths. While the isomer shifts are almost constant, we observe a very strong dependence of the quadrupole splitting with an increased agreement between calculated and experimentally observed data at shorter distances (see also Table S6). This agrees with expectations: while we do not expect the electron density at the Fe nuclei (and thus the isomer shift) to change significantly with the Fe–Fe distance, the axial compression or expansion of the distribution along the bond axis will affect the electric field gradient and thus the quadrupole splitting much more. This, in combination with the very shallow potential energy surface along the Fe–Fe bond, results in an uncertainty in the calculated quadrupole splitting parameters in the order of 0.15 mm/s. However, this uncertainty is small compared to the mean DFT error, which is of the order of 0.3 mm/s, as shown in a very recent study.^37^ These calculations thus indeed clearly support the assignment of the measured signal at δ = 0.03 mm s^–1^ to [Fe_2_(CO)_10_]^2+^. Diamagnetism of solid [Fe_2_(CO)_10_]^2+^ [AsF_6_]_2_^–^ was confirmed by SQUID measurements in the temperature range of 2–300 K (Figures S12 and S13), and the absence of an X-band EPR signal at room temperature (Figure S11).

**Table 2:** Mössbauer Parameters of Different Species Calculated at the B3LYP/ZORA-TZVPP//r^2^SCAN-3c Level as well as Experimental Results (All in mm s^–1^)

	δ_calc._	Δ*E*_calc._	δ_exp._	Δ*E*_exp._
[Fe(CO)_6_]^2+^	0.08	0.00	–0.01^b^	0.00^b^
[HFe(CO)_5_]^+^	–0.03	1.45	–0.08	1.40
[Fe(CO)_5_]^+•^	0.35	0.95	0.17^c^	0.53^c^
[Fe_2_(CO)_10_]^2+^	0.08	0.06	0.03	0.23
[Fe_2_(CO)_10_]^2+^^a^	0.08	0.15	0.03	0.23

a Calculated at the experimental Fe–Fe distance of 282 pm. This structure is about 1.0 kJ/mol above the minimum structure.

b Taken from ref (13).

c Taken from ref (14).

Interestingly, [Fe(CO)_5_]^+•^ isolated as [Al{OC(CF_3_)_3_}_4_]^−^ salt in 1,2,3,4-tetrafluorobenzene (4FB) does not dimerize^14^ for which two explanations are conceivable. A large anion size (pseudo gas phase conditions) leads, in general, to low lattice energies.^38^ Although A^2+^X^–^_2_ salts have higher lattice energies than A^+^X^–^ salts,^39^ apparently dimerization of [Fe(CO)_5_]^+•^ does not occur for the [Al{OC(CF_3_)_3_}_4_]^−^ salt in the solid state. Due to the smaller counteranions and resulting higher lattice energies for [Fe_2_(CO)_10_]^2+^ [AsF_6_]^–^_2_, observation of the dication can be explained.^40^

**Figure 4:**
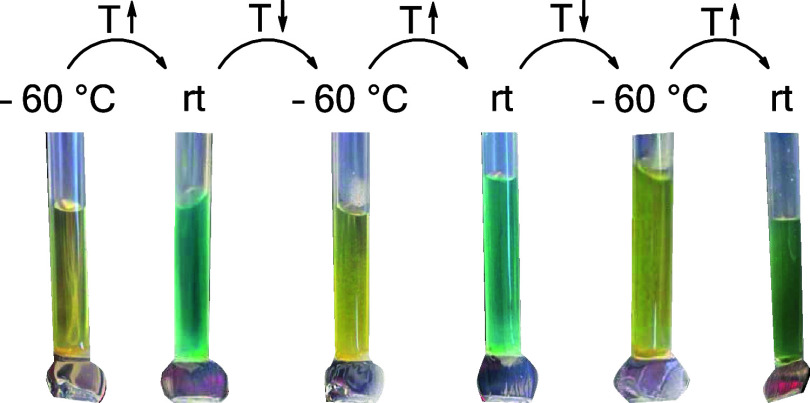
Temperature-dependent color change of [Fe_2_(CO)_10_]^2+^[AsF_6_]^–^_2_ from yellow (−60 °C) to green/blue (rt) in liquid SO_2_. The characteristic color of [Fe(CO)_5_]^+•^ in 4FB is dark green.^14^ The temperature at which the color starts to change is about −35 °C.

However, this explanation refers only to the solid state, not to the reactivity in the solution. Interestingly, the reaction of Fe(CO)_5_ in SO_2_ shows a cyclable temperature-dependent color change (Figure 4). At −60 °C, a yellow suspension is observed that starts turning green (the characteristic color of [Fe(CO)_5_]^+•^ in 4FB is dark green) at ca. −35 °C. Cooling back to −60 °C again results in a yellow suspension. This process is reversible at least four times. Both isolations at −60 °C or rt result in the yellow solid [Fe_2_(CO)_10_]^2+^[AsF_6_]^–^_2_, most likely due to the lattice energy effects mentioned above. However, EPR spectroscopy measurements of **2** in liquid SO_2_ at rt reveal the presence of a paramagnetic species with a signal similar to the one observed for solid [Fe(CO)_5_]^+•^ (Figure S11),^14^ which may be the cause of the green color change in solution. As discussed above, in the gas phase, the Fe–Fe bond is metastable and only stabilized by condensed-phase effects. At the same time, the dissociation of the dimer is strongly favored by entropy and, therefore, by a higher temperature. Using a simple Born model,^41^ the solvent part of the dimerization free energy decreases with the dielectric constant like 1/ε. SO_2_ at −60 °C has a larger dielectric constant (22.6)^42^ than 4FB at room temperature (12.7).^43^ It should be noted that this effect is mainly a temperature effect as the dielectric constant of 4FB at −60 °C can be estimated to be similar to that of SO_2_ (18.6) based on recent data.^44^ The liquid SO_2_ environment favors the formation of the dimer both entropically (due to its lower melting point) and electrostatically, offering an explanation for the dimerization in the present study and the occurrence of only the monomeric species in ref (14). Increasing the temperature of the SO_2_ solution leads to a larger entropic contribution to the dissociation-free energy and a decreased dielectric constant. Our calculations show that at −35 °C (around ε = 20.0), the electrostatic stabilization of the dimer is reduced by about 1.5 kJ/mol (see Table 1) while the value of the dissociation free energy is increased by about 5.5 kJ/mol (via the −*T*Δ*S* term). Certainly, specific interactions between solute and solvent possible in SO_2_, which cannot be modeled by a dielectric continuum model,^42,43^ will play an additional role in the process. The quantification of such effects is, however, beyond the scope of this work.

## Conclusions

To conclude, by reacting Fe(CO)_5_ with AsF_5_ in aHF, we provide the first structural characterization of [FeH(CO)_5_]^+^[AsF_6_]^−^ in the solid state, which can be regarded as one of the most acidic structurally characterized metal hydride complexes. Isolation of such highly acidic metal hydride species has proven to be elusive due to their extreme reactivity, and fully characterized complexes remain scarce. Changing to SO_2_ as the solvent resulted in the oxidation of Fe(CO)_5_. Once oxidized, the cationic species dimerizes to [Fe_2_(CO)_10_]^2+^ and two [AsF_6_]^–^. Interestingly, [Fe_2_(CO)_10_]^2+^ exhibits a shorter M–M bond length (*d*_Fe–Fe_ = 282.7(3) pm) compared to its neutral isostructural analogue Mn_2_(CO)_10_ (*d*_Mn–Mn_ = 290.3(2) pm). [Fe_2_(CO)_10_]^2+^ can be regarded as the dimerization product of the 17 VE species [Fe(CO)_5_]^+•^ and is the only example of a homoleptic dinuclear TMCC characterized by scXRD.^18b^ While a big, weakly coordinating anion ([Al{OC(CF_3_)_3_}_4_]^−^) in combination with a less polar solvent (4FB) results in the isolation of [Fe(CO)_5_]^+•^,^14^ a comparably small anion ([AsF_6_]^−^) combined with a more polar solvent (SO_2_) allows for the stabilization and isolation of the dication **2**. It therefore represents an unusual model system in which the choice of solvent and counterion determines which reactive TMCC is obtained. In addition to the already known Fe(CO)_5_ and its radical cation [Fe(CO)_5_]^+•^, it was now also possible to characterize the dimeric dication [Fe_2_(CO)_10_]^2+^ by Mössbauer spectroscopy. Quantum chemical calculations support the findings, revealing subtle changes in isomer shifts and quadrupole splittings for this unique triad of organometallic iron compounds.

## Supporting Information Available

Experimental details, spectroscopic data, structural data, and computational details. (PDF)
