# Supplementary material for: Solvent-Dependent Reactivity of Fe(CO)5 under Superacidic and Oxidative Conditions
Source: J Am Chem Soc. 2025 Jan 14;147(4):3039–46. doi: 10.1021/jacs.4c09595 (PMC11783529; doi:10.1021/jacs.4c09595)
Supplement: Supplementary file 1 — ja4c09595_si_001.pdf [file ja4c09595_si_001.pdf]

Supporting information for

Solvent-dependent Reactivity of $Fe(CO)_5$ Under Superacidic and Oxidative Conditions

Willi R. Berg,[a] Marc Reimann,[b] Robin Sievers,[a] Susanne M. Rupf,[a] Johanna Schlögl,[a] Kilian Weisser,[c] Konstantin B. Krause,[c] Christian Limberg,[c] Martin Kaupp,[b] Moritz Malischewski*[a]

[a] W. R. Berg, R. Sievers, Dr. S. M. Rupf, J. Schlögl, Dr. M. Malischewski*
Institut für Chemie und Biochemie, Anorganische Chemie
Freie Universität Berlin
Fabeckstraße 34–36, 14195 Berlin (Germany)
E-mail: moritz.malischewski@fu-berlin.de

[b] Dr. M. Reimann, Prof. Dr. M. Kaupp
Institut für Chemie
Technische Universität Berlin
Straße des 17. Juni 135, 10623 Berlin (Germany)

[c] K. Weisser, Dr. K. B. Krause, Prof. Dr. Limberg
Institut für Chemie
Humboldt-Universität zu Berlin
Brook-Taylor-Straße 2, 12489 Berlin (Germany)

Experimental details

Reactions involving *a*HF were performed in PFA (tetrafluoroethene-perfluoroalkoxyvinylcopolymer) tubes connected to a stainless-steel line. HF is a toxic and corrosive gas at room temperature. Traces of water were removed by treatment with elemental fluorine. AsF_5 is a highly toxic and corrosive gas (attention: >10 atm pressure at RT). SO_2 is a toxic gas (attention: 3 atm pressure at RT) and was dried over CaH_2. These compounds should only be handled by trained personnel with suitable protective equipment. $Fe(CO)_5$ is highly flammable and toxic. HF, SO_2 and AsF_5 were stored in stainless-steel cylinders.

Solids were handled in an argon-filled glovebox, which was also equipped with a Bruker ALPHA FTIR spectrometer with a diamond ATR attachment. Raman spectra were recorded on a Bruker MultiRAM II equipped with a low-temperature Ge detector (1064 nm). Characteristic absorptions are given in wavenumbers $\tilde{\nu}$ [cm^{-1}] and intensities are stated as vs (very strong), s (strong), m (medium) and w (weak), vw (very weak), sh. (shoulder). The software OriginPro 2017G was used to plot the data. [1]

NMR

NMR spectra were measured on a JEOL ECX 400 (400 MHz) spectrometer. The product was dissolved in anhydrous HF inside a 4mm PFA tube. A few drops of deuterated solvent were placed between this inner tube and the outer NMR glass tube. All given chemical shifts in ^1H NMR spectra are calibrated on the resonance signals of C_3H_6O contained in C_3D_6O (δ = 2.05 ppm). The ^{13}C shift was referenced to $CDCl_3$. All other spectra are device-internal calibrated relative to the resonance signal of tetramethylsilane, by the unified chemical shift scale. The given multiplicities are phenomenological, thus the actual appearance of the signals is stated and not the theoretically expected one. The following abbreviations were used and analogously combined to designate multiplicities: s (singlet), br (broad). For centrosymmetric multiplets the center and for non-symmetric multiplets the interval is stated. Evaluation of spectra was performed with Mestrelab Research MNova 7. [2]

Zero-field ^{57}Fe Mössbauer spectroscopy

Zero-field ^{57}Fe Mössbauer spectra were recorded at 14 K with a SeeCo MS6 spectrometer and a Janis CCS-850 cryostat with a CTI-Cryogenics 8200 helium compressor. The temperature was controlled with a LakeShore 335 thermocontrol. The solid samples were pressed into a PTFE sampler container. Mössbauer data was processed and simulated using the WMOSS4 program ver. 4F (www.wmoss.org). Isomeric shifts are referenced to alpha-iron at room temperature.

SQUID

Magnetic measurements were performed with a QuantumDesign MPMS3 SQUID magnetometer. The samples were prepared in a glovebox in VSM powder capsules which were sealed with a piece of Teflon tape. Both the capsules and the Teflon tape were dried in a Schlenk flask under vacuum at 110 °C for five days. A brass sample holder was used. The measurement was carried out in VSM mode from 2 K to 300 K in a magnetic field of 7 T after cooling down at zero field. A background correction was applied by subtracting the magnetic moments of an empty capsule sealed with a piece of Teflon tape using the same measurement

sequence as for the sample. A diamagnetic correction was performed using Pascal's constants.[3]

EPR

The EPR spectrum of $[Fe_2(CO)_{10}]^{2+}[AsF_6]^-{}_2$ was recorded in the X-band at 298 K and 77 K with a Magnetech MS 5000 spectrometer. The samples were sealed under reduced pressure inside a 4 mm quartz tube.

X-Ray Diffraction (XRD)

X-Ray data was measured on a BRUKER X8 KAPPA APEX II diffractometer. The Data was collected at 100-105 K using graphite-monochromated Mo Kα radiation (λα = 0.71073 Å). The strategy for the data collected on was evaluated by using the Smart software. The strategy for the data collection was gathered by the standard "ψ-ω scan techniques" and were scaled and reduced using Saint+ software. The structures were solved by using Olex2[4] and XT[5] structure solution program using Intrinsic Phasing and refined with the XL refinement package[6] using at Least Squares minimization. Crystal drawings were generated with Diamond[7] and POVRay[8].

Experimental part:

$[FeH(CO)_5]^+[AsF_6]^-$

In a 8 mm PFA tube anhydrous HF (1 mL) and AsF_5 (140 mg, 0.824 mmol, 2.78 eq.) were condensed onto $Fe(CO)_5$ (58.0 mg, 0.296 mmol, 1.00 eq.). The reaction mixture was warmed up to –60 °C forming a colorless insoluble precipitate. After several minutes of shaking at –60 °C all volatiles were removed under reduced pressure at –60 °C, resulting in a colorless solid (85.5 mg, 0.222 mmol, 75%). Colorless crystals of $[FeH(CO)_5]^+[AsF_6]^-$ were obtained by recrystallization of the solid in anhydrous HF upon slow cooling from rt to −70 °C.

IR $\tilde{\nu}$ [cm^{-1}] = 2124 (vs), 2095 (sh.), 784 (m), 695 (vs), 673 (sh.), 612 (s), 585 (vs), 537 (sh.), 496 (sh.), 431 (m).

Raman $\tilde{\nu}$ [cm^{-1}] = 2199 (vs), 2156 (vs), 2139 (vs), 1894 (m), 790 (w), 683 (m), 382 (w), 369 (vw), 134 (m), 120 (sh.) 78 (sh).

^{1}H NMR (400 MHz, C_3D_6O/*a*HF, 298 K): δ [ppm] = –8.40 (s, 1H).

^{13}C NMR (151 MHz, $CDCl_3$/*a*HF, 298 K): δ [ppm] = 192.0 (s, $C_{eq.}$), 190.3 (s, $C_{ax.}$).

^{19}F NMR (377 MHz, C_3D_6O/HF, 298 K): δ [ppm] = –198.5 (s, HF), –70.2 (br)

Zero-field ^{57}Fe Mössbauer (solid, 14 K): isomer shift δ [mm s^{-1}] = –0.08; QS ΔE [mm s^{-1}] = 1.40.

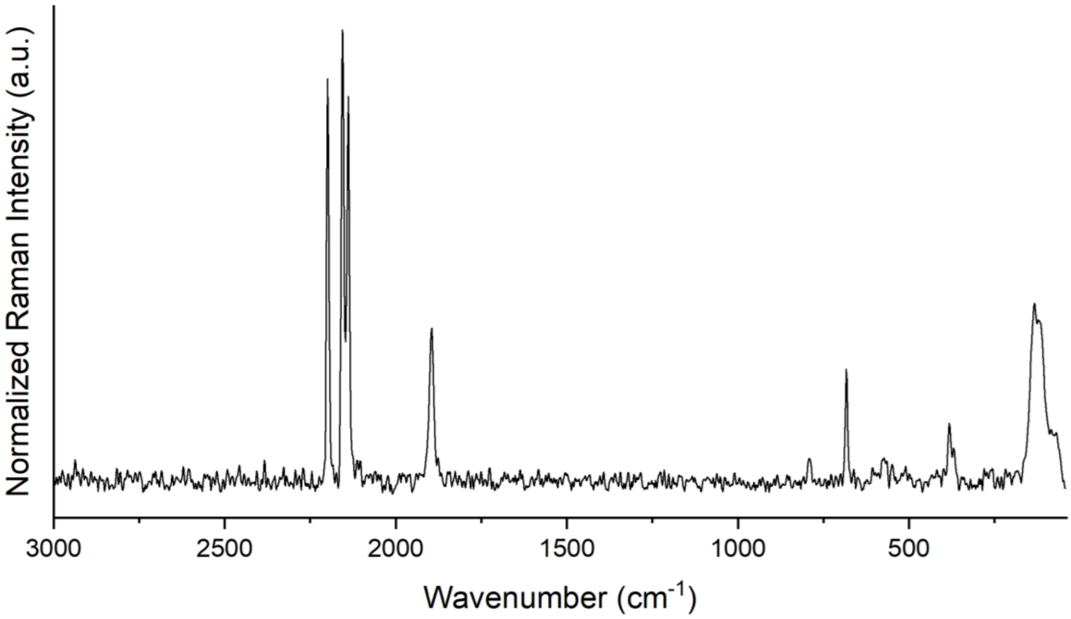

Figure S1. Raman spectrum (bulk, 298 K) of $[FeH(CO)_5]^+[AsF_6]^-$.

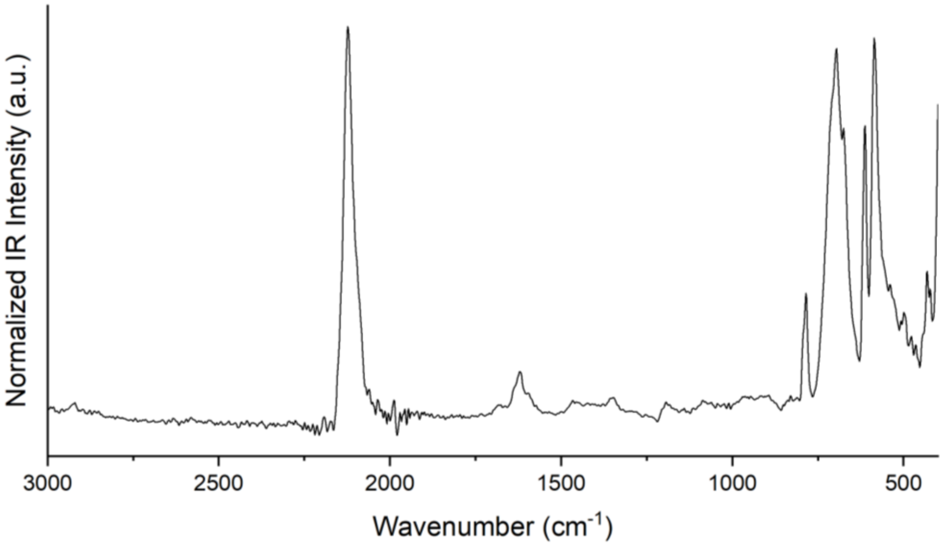

Figure S2. IR spectrum (ATR, bulk, 298 K) of $[FeH(CO)_5]^+[AsF_6]^-$.

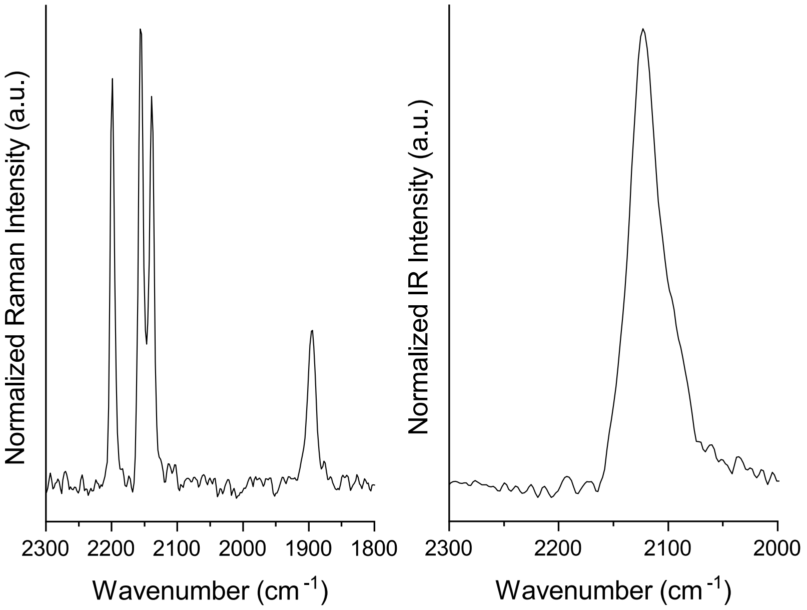

Figure S3. The carbonyl region of $[FeH(CO)_5]^+[AsF_6]^-$ of the Raman (left, corresponding full spectrum Figure S1) and IR (right, corresponding full spectrum Figure S2) spectrum.

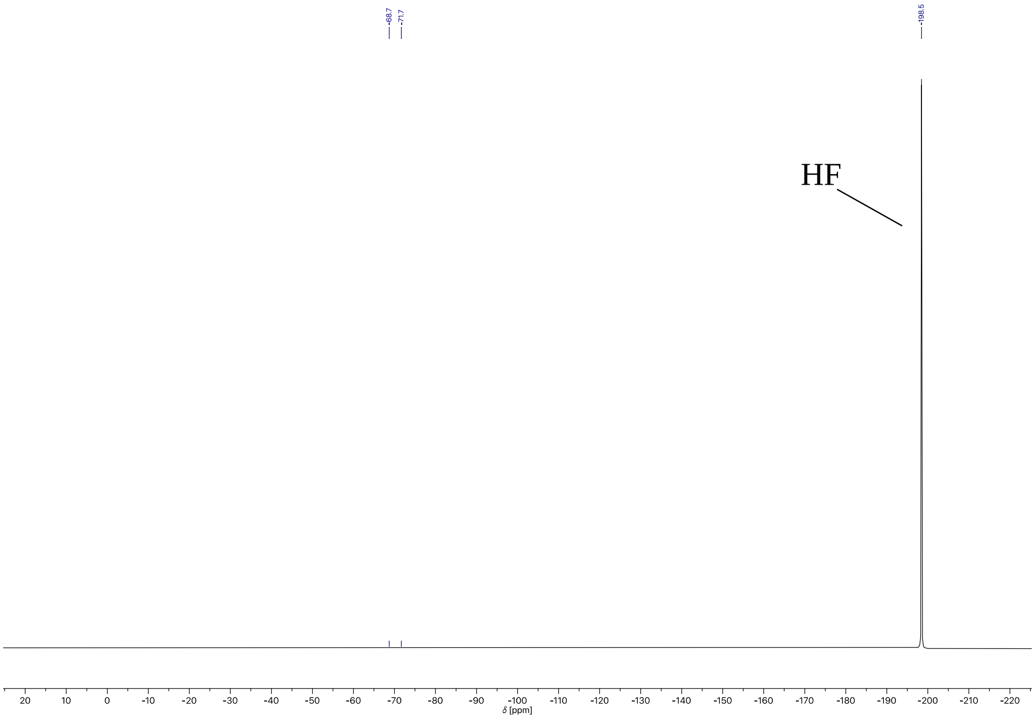

Figure S4. ^{19}F NMR (377 MHz, *a*HF/C_3D_6O, rt) of $[FeH(CO)_5]^+[AsF_6]^-$. $[FeH(CO)_5]^+[AsF_6]^-$ was dissolved in *a*HF inside a PFA tube. The PFA tube was subsequently put in a glass NMR tube and surrounded by C_3D_6O.

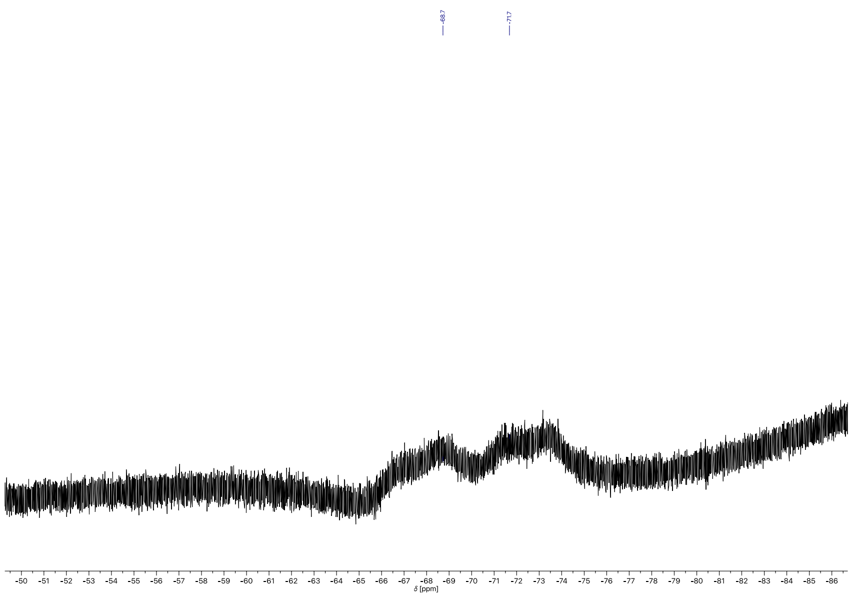

Figure S5. Broad saddle-shaped signal in the ^{19}F NMR (377 MHz, *a*HF/C_3D_6O, rt) of $[FeH(CO)_5]^+[AsF_6]^-$, corresponding to the $[AsF_6]^-$ counter ion, as a result of partial quadrupole-collapse of the $^1J(^{75}As–^{19}F)$ coupling.[9]

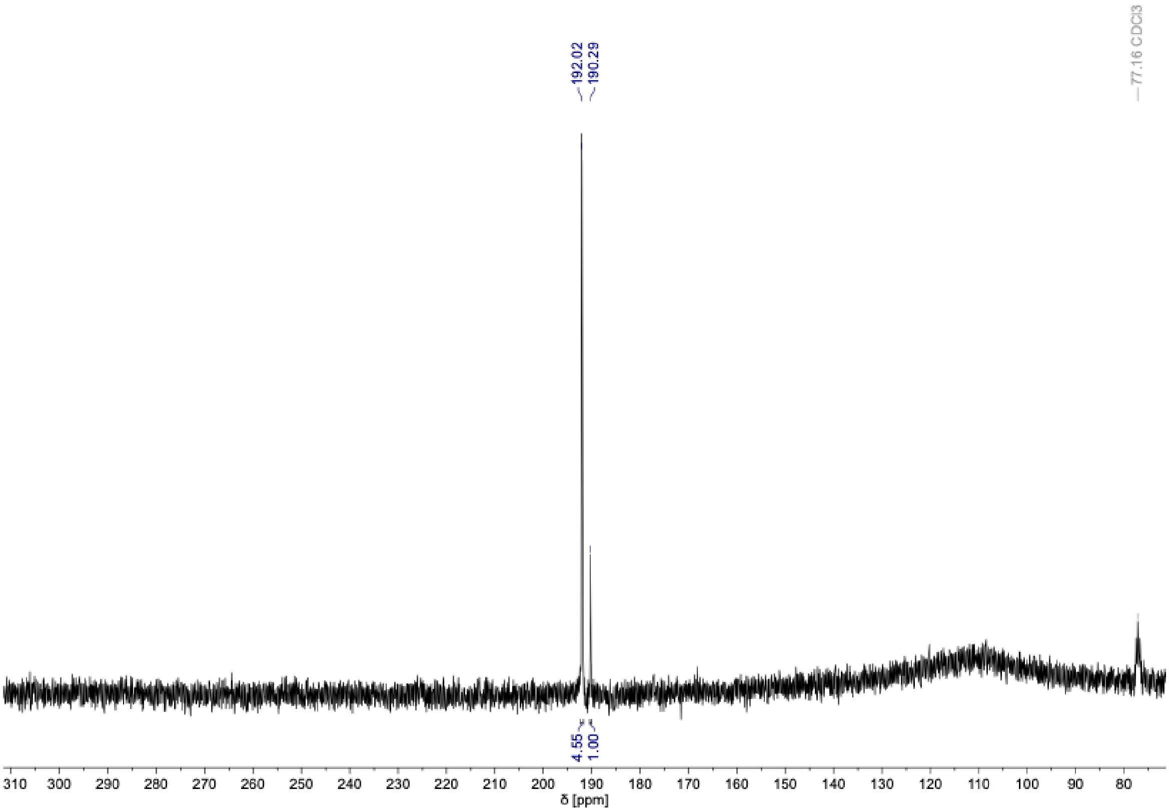

Figure S6. ^{13}C NMR (151 MHz, $CDCl_3$/*a*HF, 298 K) of $[FeH(CO)_5]^+[AsF_6]^-$. $[FeH(CO)_5]^+[AsF_6]^-$ was dissolved in *a*HF inside a PFA tube. The PFA tube was subsequently put in a glass NMR tube and surrounded by $CDCl_3$.

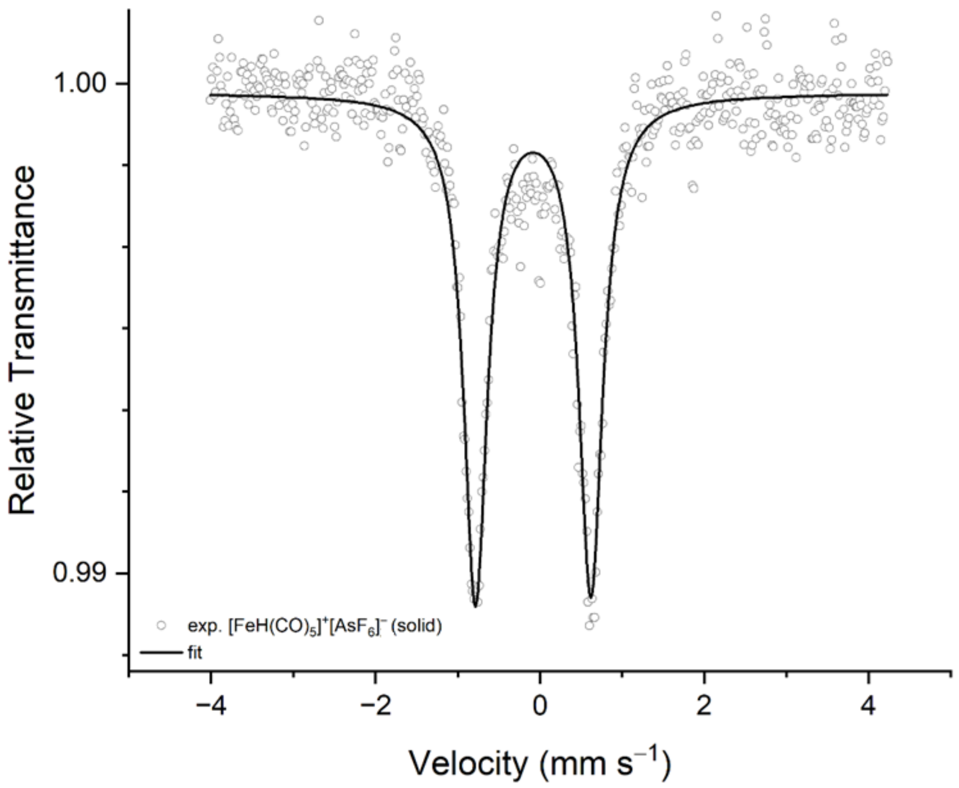

Figure S7. Zero-field ^{57}Fe Mössbauer spectrum of $[FeH(CO)_5]^+[AsF_6]^-$. The solid black line represents the numerical fit of the measured experimental data indicated by the grey circle.

$[Fe_2(CO)_{10}]^{2+}[AsF_6]^-_2$

In a 8 mm PFA tube $Fe(CO)_5$ (90.0 mg, 0.459 mmol, 1.00 eq.) was reacted with AsF_5 (300 mg, 1.77 mmol, 3.86 eq.) in SO_2 (1 mL) resulting in a yellow precipitate with a yellow solution, which turns green-blue at rt. Color change back to yellow is observed when cooled back to –60 °C. After several minutes of rigorous shaking at –60 °C a yellow solid (280 mg, 0.364 mmol, 79%) is obtained after removal of all volatiles at –60 °C. Yellow needles of $[Fe_2(CO)_{10}]^{2+}[AsF_6]^-_2 \cdot 2\ SO_2$ were obtained by recrystallization of the solid in SO_2 upon slow cooling from rt to –70 °C.

IR $\tilde{\nu}$ [cm^{-1}] = 2208 (w), 2160 (s), 2127 (m), 2107 (sh.), 956 (w), 878 (s), 833 (sh.), 788 (vw), 704 (vs), 650 (m), 612 (sh.), 578 (vs).

Raman $\tilde{\nu}$ [cm^{-1}] = 2207 (vs), 2184 (s), 2170 (sh), 2156 (sh.), 887 (w), 652 (w), 378 (m), 123 (vs), 74 (sh.).

Zero-field ^{57}Fe Mössbauer (solid, 14 K): isomer shift δ [mm s^{-1}] = 0.03; QS ΔE [mm s^{-1}] = 0.23.

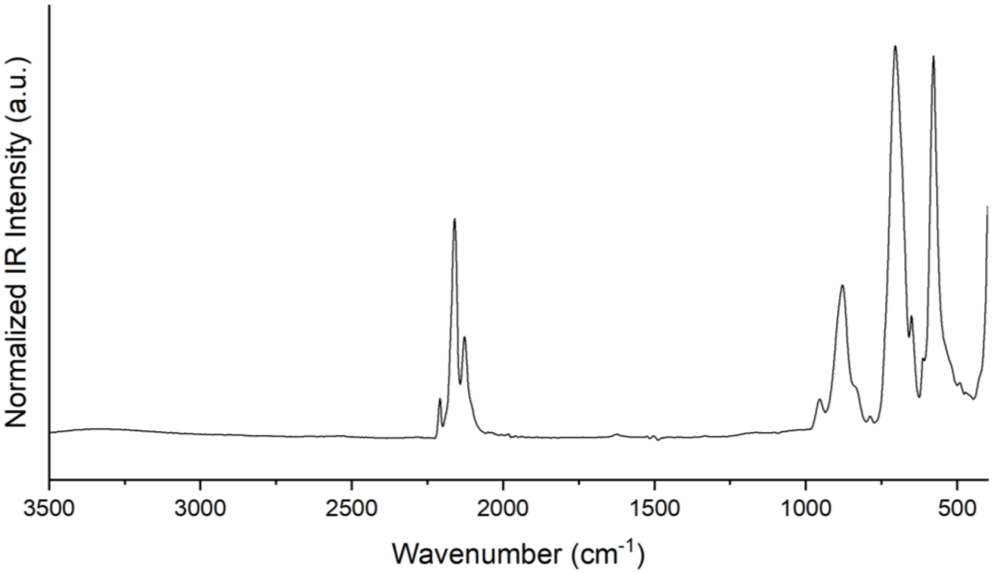

Figure S8. IR spectrum (ATR, bulk, 298 K) of $[Fe_2(CO)_{10}]^{2+}[AsF_6]^{-}_2$.

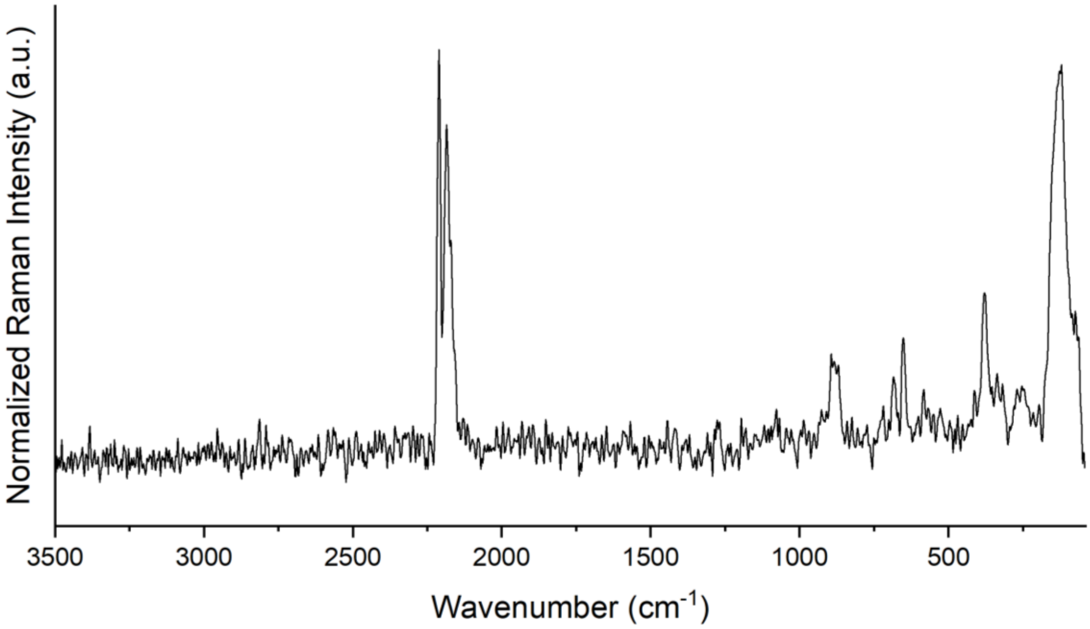

Figure S9. Raman spectrum (bulk, 298 K) of $[Fe_2(CO)_{10}]^{2+}[AsF_6]^{-}_2$.

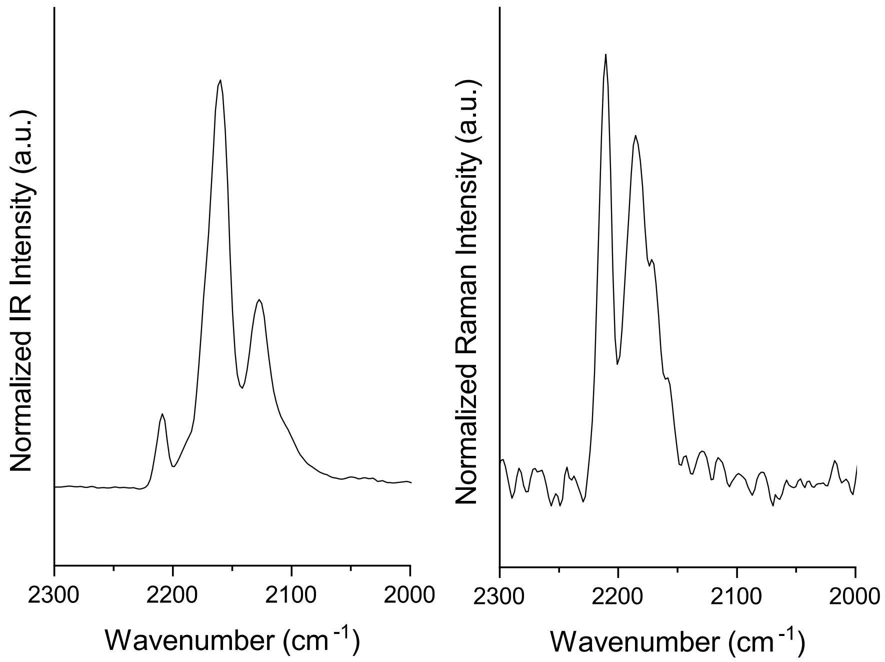

Figure S10. The carbonyl region of $[Fe_2(CO)_{10}]^{2+}[AsF_6]^-_2$ of the IR (left, corresponding full spectrum Figure S8) and Raman (right, corresponding full spectrum Figure S9) spectrum.

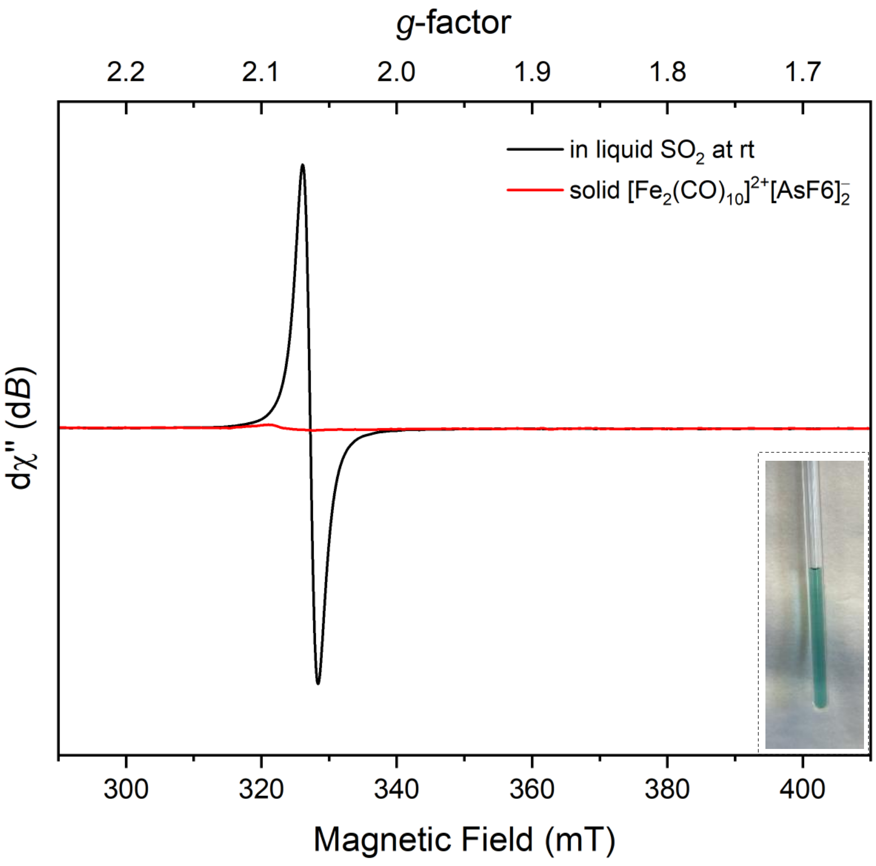

Figure S11. Continuous wave EPR spectrum (9.40 GHz) of solid $[Fe_2(CO)_{10}]^{2+}[AsF_6]^-_2$ (red)

and in liquid SO_2 at rt (black). Inset shows green solution after dissolving $[Fe_2(CO)_{10}]^{2+}[AsF_6]^{-}_2$ at rt.

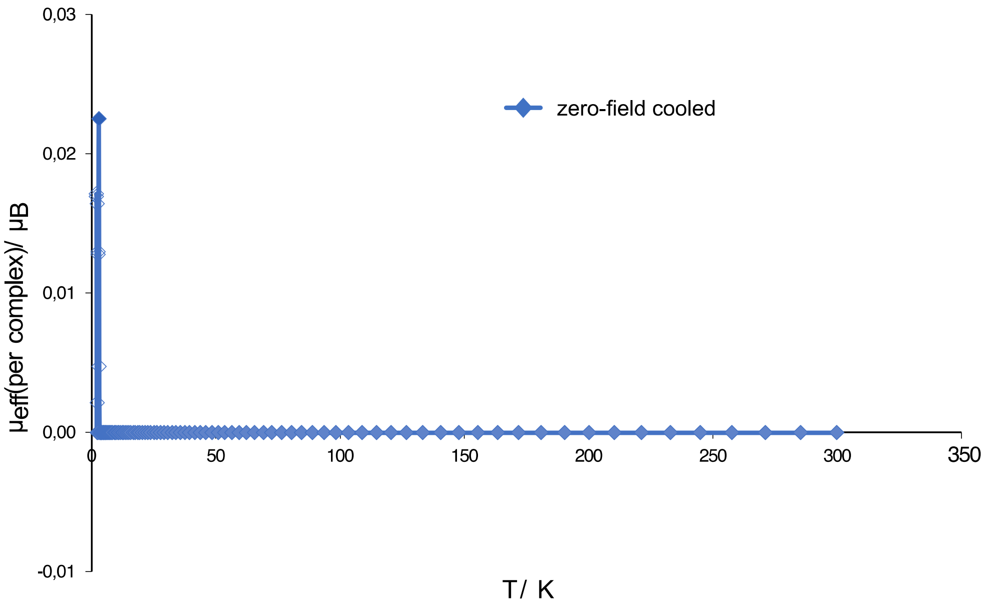

Figure S12. SQUID measurement of solid $[Fe_2(CO)_{10}]^{2+}[AsF_6]^{-}_2$ from 2 to 300 K at constant magnetic field (7 T) after zero-field cooling.

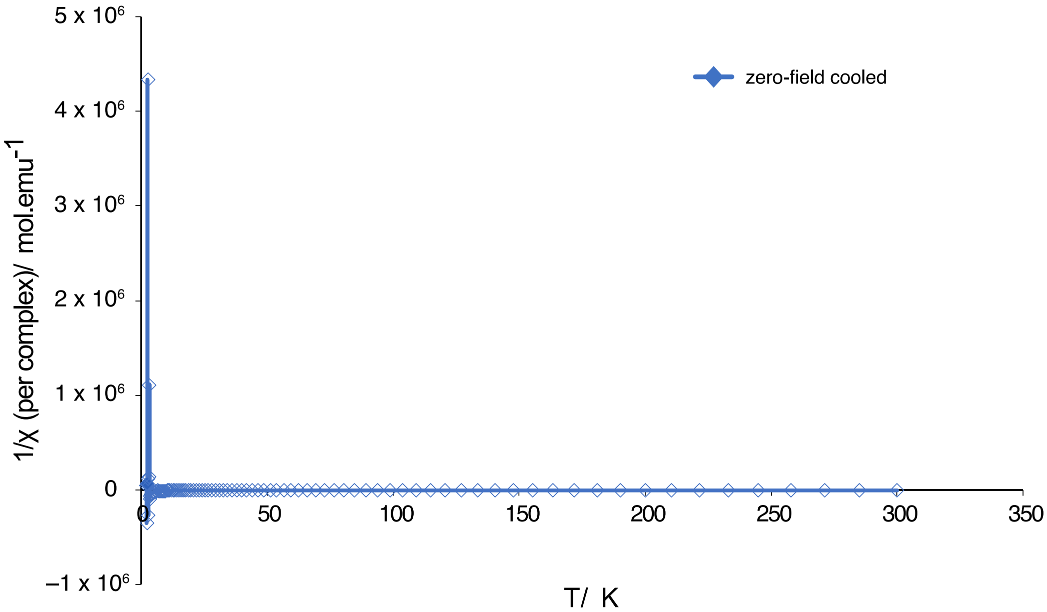

Figure S13. SQUID measurement of solid $[Fe_2(CO)_{10}]^{2+}[AsF_6]^{-}_2$ from 2 to 300 K at constant magnetic field (7 T) after zero-field cooling.

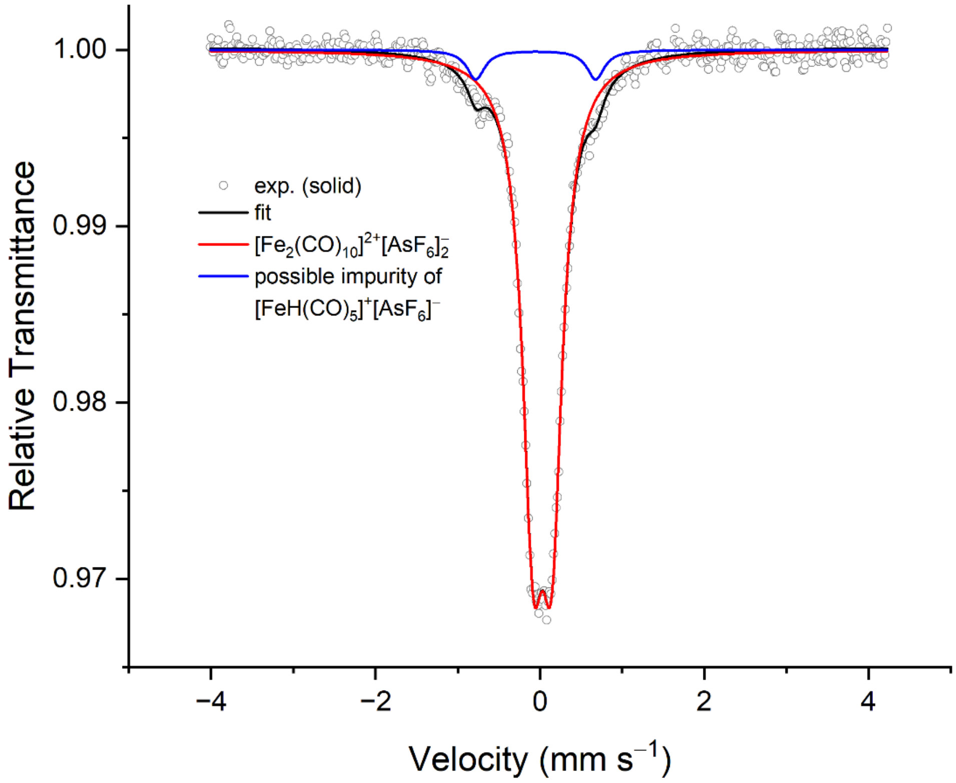

Figure S14. Zero-field Mössbauer spectrum of solid $[Fe_2(CO)_{10}]^{2+}[AsF_6]^{-}_2$ (experimental data indicated by the grey circles). A slight broadening at the basis of the main signal (red line) might be attributable to a second species. Including this into the experimental fit (solid black line) leads to an integral of 6% and the parameters $\delta = -0.05$ mm s^{-1} / $\Delta E = 1.46$ mm s^{-1}, so that this signal (blue line) may originate in $[FeH(CO)_5]^{+}[AsF_6]^{-}$ (isomer shift: $\delta = -0.08$ mm s^{-1} QS: $\Delta E = 1.40$ mm s^{-1}). Consideration in the fit did not lead to an improvement of the R value, though (without second species: R = 0.747, with second species: R = 0.556, Figure S14), so that some uncertainty remains with this assessment. However, given that $[FeH(CO)_5]^{+}[AsF_6]^{-}$ had been found to cocrystallize with $[Fe_2(CO)_{10}]^{2+}[AsF_6]^{-}_2$ and that the slight deviations in the parameters may be due the lack of resolution, we find the presence of 6% $[FeH(CO)_5]^{+}[AsF_6]^{-}$ a likely explanation for the broadening of the main signal base.

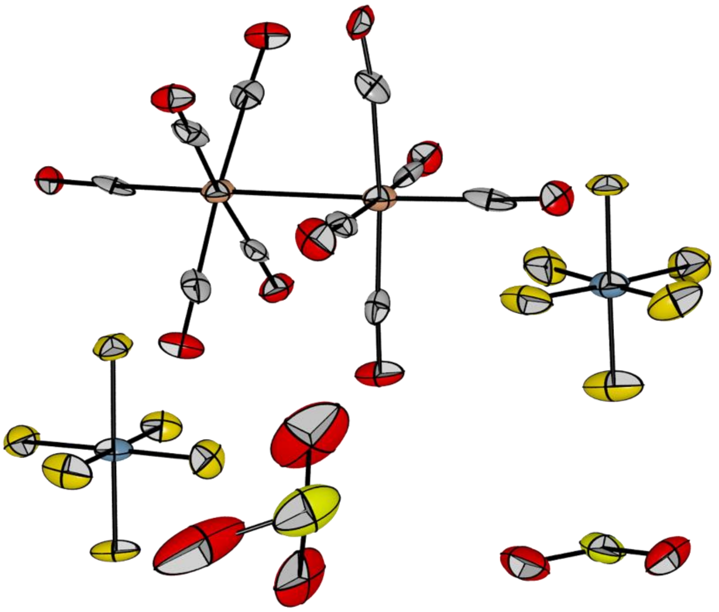

Figure S15. Molecular structure in the solid-state of $[Fe_2(CO)_{10}]^{2+}[AsF_6]^-{}_2 \cdot 2\ SO_2$. Color code: orange = iron, grey = carbon, red = oxygen, light grey = hydrogen, blue = arsenic, dark yellow = fluorine, bright yellow = sulfur. One SO_2 molecule is disordered.

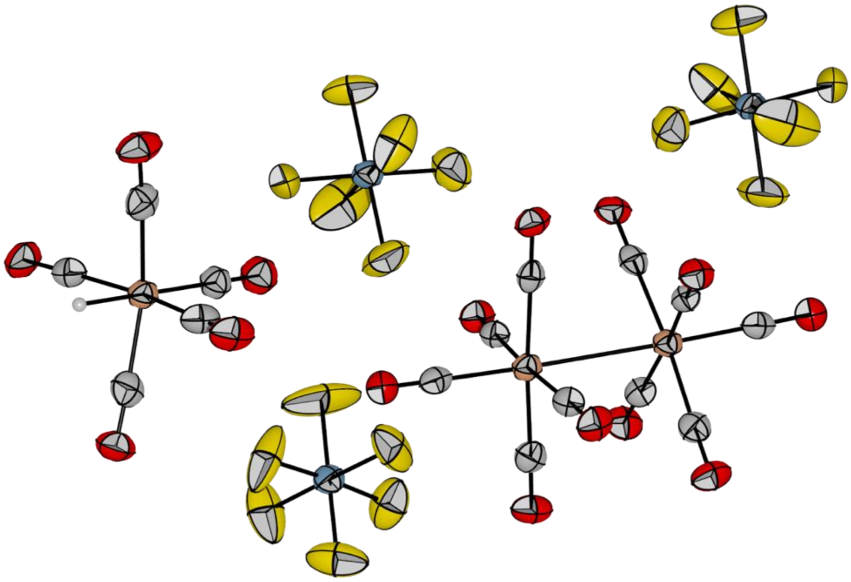

Figure S16. Molecular structure in the solid-state of $[Fe_2(CO)_{10}]^{2+}{}_2[FeH(CO)_5]^+[AsF_6]^-{}_5$. Color code: orange = iron, grey = carbon, red = oxygen, light grey = hydrogen, blue = arsenic, yellow = fluorine.

Quantum Chemical Calculations

Initial structure optimizations followed by harmonic vibrational frequency calculations of $[FeH(CO)_5]^+$ and $[Fe_2(CO)_{10}]^{2+}$ complexes were performed at the B3LYP-D3(BJ)/def2-TZVPP level of theory using the Gaussian program (version 16 A.03).[10] All calculations employed default settings. Frequencies are scaled with 0.968 according to Duncan *et al.*[11] Additional structure optimizations were performed at the r^2SCAN-3c level[12] using the ORCA program, version 5.0.2.[13] All calculations included a continuum solvent model (CPCM with

parametrization for CH_2Cl_2 as a solvent with intermediate polarity) to approximately account for the dielectric effects of different solvents or the crystal environment, a tight DFT grid (DefGrid3), and tight optimization criteria (VeryTightSCF and TightOpt). Mössbauer parameters were calculated at the B3LYP/DKH-def2-TZVP [14] level following the protocol of Ref. [15]. Scalar relativistic effects were included using the second order Douglas Kroll Hess Hamiltonian.[16] For the iron atom, CP(PPP) basis sets[17] and an increased radial integration grid (SpecialGridIntAcc 7) were employed.

Energy decomposition analyses and Extended transition-state analyses with natural orbitals for chemical valence (ETS-NOCV) were performed at the BP86-D4/TZP [18] level using the ADF engine of the AMS program package, release 2023.1.[19] Calculations with COSMO employed the solvent radius of CH_2Cl_2 in the cavity construction and the dielectric constants discussed in the main text.

$[FeH(CO)_5]^+$

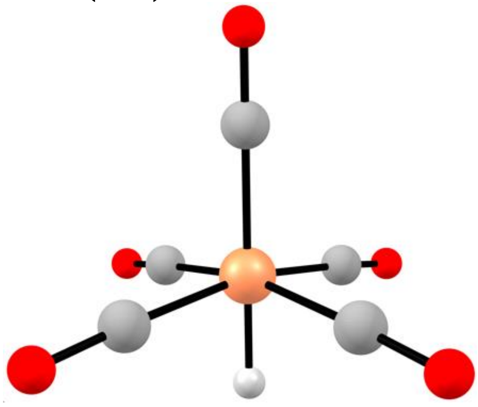

Figure S17. Calculated (B3LYP-D3(BJ)/def2-TZVPP) minimum structure of $[FeH(CO)_5]^+$. Color code: orange = iron, grey = carbon, red = oxygen, white = hydrogen.

Energy = –1831.07194727 Hartree

Table S1. Calculated (B3LYP-D3(BJ)/def2-TVZPP) atomic coordinates of the minimum structure of $[FeH(CO)_5]^+$.

Fe	0.000001000	-0.000080000	-0.167292000
C	0.671184000	-1.729177000	-0.381536000
C	-0.000275000	0.000538000	1.738205000
O	1.071910000	-2.760908000	-0.566511000
O	-0.000440000	0.000948000	2.860339000
C	-1.729161000	-0.671271000	-0.381421000
C	1.729248000	0.670938000	-0.381356000
O	-2.761010000	-1.071857000	-0.566013000
C	-0.671252000	1.728934000	-0.382399000
O	-1.071464000	2.760811000	-0.567648000
O	2.761152000	1.071399000	-0.565909000
H	0.000326000	-0.000855000	-1.673440000

Table S2. Calculated (B3LYP-D3(BJ)/def2-TZVPP) vibrational frequencies and IR (left) and Raman (right) intensities of $[FeH(CO)_5]^+$ with their corresponding intensities. Frequencies are scaled with 0.968 according to Duncan *et al.*[11]

**IR**		**Raman**	
**Wavenumber [cm^{-1}]**	**Intensity [KM/mol]**	**Wavenumber [cm^{-1}]**	**Intensity [A^4/AMU]**
54.3	0.0	54.3	0.0
80.0	0.0	80.0	3.2
80.0	0.0	80.0	3.2
97.8	0.0	97.8	5.5
104.6	0.7	104.6	0.7
104.6	0.7	104.6	0.7
112.7	0.6	112.7	0.4
329.7	2.4	329.7	0.8
329.7	2.4	329.7	0.8
343.9	0.0	343.9	0.0
353.9	1.7	353.9	7.9
369.7	0.0	369.7	0.2
386.3	2.8	386.3	1.3
406.4	17.3	406.4	0.1
406.4	17.3	406.4	0.1
463.8	0.0	463.8	0.1
496.3	2.6	496.3	0.2
496.3	2.6	496.3	0.2
538.3	0.0	538.3	0.0
580.7	78.6	580.7	0.5
580.7	78.7	580.7	0.5
607.8	104.7	607.8	0.0
797.7	47.5	797.7	4.0
797.7	47.5	797.7	4.0
1903.9	7.2	1903.9	118.7
2150.9	770.8	2150.9	6.5
2150.9	770.8	2150.9	6.5
2156.4	397.8	2156.4	114.5
2161.6	0.0	2161.6	191.3
2203.6	158.9	2203.6	158.9

$[Fe_2(CO)_{10}]^{2+}$

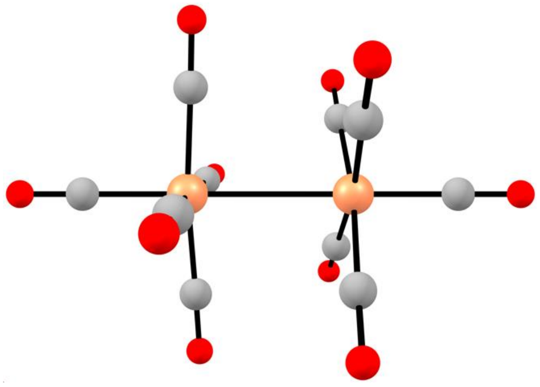

Figure S18. Calculated (B3LYP-D3(BJ)/def2-TZVPP) minimum structure of $[Fe_2(CO)_{10}]^{2+}$. Color code: orange = iron, grey = carbon, red = oxygen.

Energy: –3660.86130752 Hartree

Table S3. Calculated (B3LYP-D3(BJ)/def2-TZVPP) atomic coordinates of the minimum structure of $[Fe_2(CO)_{10}]^{2+}$.

Fe	1.485613000	0.000068000	-0.000006000
Fe	-1.485902000	-0.000090000	0.000076000
C	-3.363802000	-0.000632000	0.000216000
C	-1.379743000	1.119436000	1.502066000
C	1.379492000	1.853707000	0.271029000
O	-4.483487000	-0.000970000	0.000300000
C	3.363505000	0.000727000	-0.000187000
O	1.333031000	2.963829000	0.433172000
O	-1.332335000	1.790090000	2.401401000
O	4.483191000	0.001181000	-0.000317000
C	1.379179000	0.271009000	-1.853593000
C	-1.379021000	-1.502213000	1.119290000
C	1.379285000	-0.271032000	1.853541000
O	1.332159000	0.433180000	-2.963694000
C	1.380492000	-1.853687000	-0.270905000
O	-1.332582000	-1.789835000	-2.401598000
O	1.334560000	-2.963824000	-0.433057000
O	1.331588000	-0.433493000	2.963570000
C	-1.380239000	1.502161000	-1.119215000
O	-1.331115000	-2.401929000	1.789405000
O	-1.333804000	2.401791000	-1.789539000
C	-1.379504000	-1.119405000	-1.502067000

Table S4. Calculated (B3LYP-D3(BJ)/def2TZVPP) IR frequencies of $[Fe_2(CO)_{10}]^{2+}$ with their corresponding intensities. Frequencies are scaled with 0.968 according to Duncan *et al.*[11]

**IR** Wavenumber [cm^{-1}]	**Intensity [KM/mol]**
38.4	0.0
56.6	0.1
56.6	0.1

61.4	0.0
61.4	0.0
76.8	0.0
76.9	0.0
84.9	0.0
84.9	0.0
96.7	1.1
96.8	1.1
98.6	0.0
98.6	0.0
102.0	0.0
106.0	0.9
106.0	0.9
109.1	0.0
109.1	0.0
117.3	0.4
204.8	0.0
340.9	0.0
342.1	0.0
348.4	50.3
355.1	0.0
355.1	0.0
362.3	0.0
362.3	0.0
363.7	0.0
367.6	1.1
367.6	1.1
379.9	8.1
398.8	0.0
409.7	28.3
409.7	28.2
414.6	0.0
414.6	0.0
438. 8	0.0
439.0	0.0
495.8	0.0
495.9	0.0
508.1	7.4
508.2	7.4
526.2	0.0
526.3	0.0
586.8	0.0
586.8	0.0
589.1	141.1
589.1	141.2
589.2	440.8
616.3	0.0
2132.6	0.0
2132.7	0.0
2142.3	160.8

2149.1	1224.0
2149.1	1224.2
2150.6	0.0
2150.6	0.0
2171.1	0.0
2179.4	1085.6
2206.7	0.0

Table S5. Calculated (B3LYP-D3(BJ)/def2-TZVPP) Raman frequencies of $[Fe_2(CO)_{10}]^{2+}$ with their corresponding intensities. Frequencies are scaled with 0.968 according to Duncan *et al.* [11]

**Raman**<br>**Wavenumber [cm^{-1}]**	**Intensity [A^4/AMU]**
38.4	0.0
56.6	0.0
56.6	0.0
61.4	2.8
61.4	2.8
76.8	0.5
76.9	0.5
84.9	4.8
84.9	4.8
96.7	0.0
96.8	0.0
98.6	3.6
98.6	3.7
102.0	6.1
106.0	0.0
106.0	0.0
109.1	1.2
109.1	1.2
117.3	0.0
204.8	23.7
340.9	0.0
342.1	0.0
348.4	0.0
355.1	0.5
355.1	0.5
362.3	0.5
362.3	0.5
363.7	19.7
367.6	0.0
367.6	0.0
379.9	0.0
398.8	19.5
409.7	0.0
409.7	0.0
414.6	0.6
414.6	0.6
438. 8	1.4

439.0	1.4
495.8	1.3
495.9	1.3
508.1	0.0
508.2	0.0
526.2	0.0
526.3	0.0
586.8	0.8
586.8	0.8
589.1	0.0
589.1	0.0
589.2	0.0
616.3	16.1
2132.6	86.7
2132.7	86.8
2142.3	0.0
2149.1	0.0
2149.1	0.0
2150.6	147.5
2150.6	147.4
2171.1	427.4
2179.4	0.0
2206.7	320.8

Table S6. Relative energies and Mössbauer parameters of $[Fe_2(CO)_{10}]^{2+}$ using partially relaxed structures at different fixed Fe−Fe distances. Structures were optimized at the r^{2}SCAN-3c level.

Distance [Å]	Relative Energies [kJ/mol]	δ [mm/s]	ΔE [mm/s]
2.75	4.02	0.09	−0.247
2.76	3.45	0.09	−0.231
2.77	2.92	0.09	−0.216
2.78	2.44	0.09	−0.202
2.79	2.01	0.08	−0.189
2.80	1.62	0.08	−0.174
2.81	1.28	0.08	−0.161
2.82	0.98	0.08	−0.148
2.83	0.72	0.08	−0.135
2.84	0.50	0.08	−0.122
2.85	0.33	0.08	−0.111
2.86	0.19	0.08	−0.099
2.87	0.09	0.08	−0.087
2.88	0.02	0.08	−0.076
2.89	0.00	0.08	−0.065
2.90	0.01	0.08	−0.054
2.91	0.06	0.08	−0.044
2.92	0.14	0.08	−0.033
2.93	0.25	0.08	−0.023
2.94	0.40	0.08	−0.014
2.95	0.58	0.08	−0.004

Table S7. Gas phase proton affinities (PA) of $Fe(CO)_5$, $Re(CO)_5H$, and organic bases tetrahydrofurane and acetonitrile in kJ mol^{-1} calculated on B3LYP-D3(BJ)/def2-TZVPP level of theory.

	PA [kJ mol^{-1}]
$Fe(CO)_5$	815
$Re(CO)_5H$	835
Tetrahydrofurane	832
Acetonitrile	789

Crystallographic Data

Table S8. Crystallographic data of $[FeH(CO)_5]^+[AsF_6]^-$.

Identification code	2365191
Empirical formula	$C_5HAsF_6FeO_5$
Formula weight	385.83
Temperature/K	100.00
Crystal system	monoclinic
Space group	C2/c
a/Å	26.5159(8)
b/Å	6.8338(3)
c/Å	13.3407(5)
α/°	90
β/°	116.8840(10)
γ/°	90
Volume/Å^3	2156.13(14)
Z	8
ρ_{calc}g/cm^3	2.377
μ/mm^{-1}	4.541
F(000)	1472.0
Crystal size/mm^3	0.1 × 0.1 × 0.1
Radiation	MoKα (λ = 0.71073)
2Θ range for data collection/°	6.118 to 56.602
Index ranges	$-35 \le h \le 35$, $-9 \le k \le 9$, $-17 \le l \le 17$
Reflections collected	22753
Independent reflections	2669 [R_{int} = 0.0380, R_{sigma} = 0.0208]
Data/restraints/parameters	2669/0/170
Goodness-of-fit on F^2	1.125
Final R indexes [I>=2σ (I)]	R_1 = 0.0255, wR_2 = 0.0561
Final R indexes [all data]	R_1 = 0.0311, wR_2 = 0.0587
Largest diff. peak/hole / e Å^{-3}	0.63/-0.80

Table S9. Crystallographic data of $[Fe_2(CO)_{10}]^{2+}[AsF_6]^{-}{}_2 \cdot 2\ SO_2$.

Identification code	2365192
Empirical formula	$As_2C_{10}F_{12}Fe_2O_{14}S_2$
Formula weight	897.76
Temperature/K	100.00
Crystal system	orthorhombic
Space group	$P2_12_12_1$
a/Å	19.0462(15)
b/Å	8.9746(7)
c/Å	15.7905(11)
α/°	90
β/°	90
γ/°	90
Volume/Å^3	2699.7(4)
Z	4
ρ_{calc}g/cm^3	2.209
μ/mm^{-1}	3.81
F(000)	1720.0
Crystal size/mm^3	0.29 × 0.18 × 0.07
Radiation	MoKα (λ = 0.71073)
2Θ range for data collection/°	4.2 to 50.4
Index ranges	$-22 \leq h \leq 22, -10 \leq k \leq 10, -18 \leq l \leq 18$
Reflections collected	72702
Independent reflections	4840 [R_{int} = 0.1241, R_{sigma} = 0.0417]
Data/restraints/parameters	4840/2/392
Goodness-of-fit on F^2	1.20
Final R indexes [I>=2σ (I)]	R_1 = 0.0586, wR_2 = 0.1378
Final R indexes [all data]	R_1 = 0.0991, wR_2 = 0.1719
Largest diff. peak/hole / e Å^{-3}	1.21/-0.77
Flack parameter	0.50(4)
BASF	0.50470

Table S10. Crystallographic data of $[Fe_2(CO)_{10}]^{2+}{}_2[FeH(CO)_5]^{+}[AsF_6]^{-}{}_5$.

Identification code	2365193
Empirical formula	$C_{25}HAs_5F_{30}Fe_5O_{25}$
Formula weight	1925.11
Temperature/K	100
Crystal system	tetragonal
Space group	P4/ncc
a/Å	19.5353(5)

b/Å	19.5353(5)
c/Å	14.2121(7)
α/°	90
β/°	90
γ/°	90
Volume/Å^3	5423.7(4)
Z	4
ρ_{calc}g/cm^3	2.358
μ/mm^{-1}	4.513
F(000)	3664.0
Crystal size/mm^3	0.1 × 0.1 × 0.1
Radiation	MoKα (λ = 0.71073)
2Θ range for data collection/°	4.17 to 52.732
Index ranges	$-23 \leq h \leq 24$, $-24 \leq k \leq 21$, $-14 \leq l \leq 17$
Reflections collected	25832
Independent reflections	2778 [R_{int} = 0.0488, R_{sigma} = 0.0252]
Data/restraints/parameters	2778/0/208
Goodness-of-fit on F^2	1.129
Final R indexes [I>=2σ (I)]	R_1 = 0.0471, wR_2 = 0.1129
Final R indexes [all data]	R_1 = 0.0739, wR_2 = 0.1395
Largest diff. peak/hole / e Å^{-3}	1.54/-1.30

References

[1] Origin(Pro), *Version 2016*, OriginLab Corporation: Northhampton, Massachusetts, USA, 2016.

[2] M. R. Willcott, *J. Am. Chem. Soc.* **2009**, *131*, 13180.

[3] G. A. Bain, J. F. Berry, *J. Chem. Educ.* **2008**, *85*, 532.

[4] O. V. Dolomanov, L. J. Bourhis, R. J. Gildea, J. A. K. Howard, H. Puschmann, *J. Appl. Cryst.* **2009**, *42*, 339-341.

[5] G. Sheldrick, *Acta Cryst. A* **2015**, *71*, 3-8.

[6] G. Sheldrick, *Acta Cryst. A* **2008**, *64*, 112-122.

[7] K. Brandenburg, *Diamond (3.2 ed)*, Crystal Impact GbR, Bonn, 2014.

[8] Persistence of Vision Pty. Ltd. Persistence of Vision Raytracer. Ltd., Persistence of Vision Pty. 2004. Retrieved from http://www.povray.org/download/.

[9] G. L. Smith, H. P. A. Mercier, G. J. Schrobilgen, *Inorg. Chem.* **2007**, *46*, 1369-1378.

[10] M. J. Frisch, G. W. Trucks, H. B. Schlegel, G. E. Scuseria, M. A. Robb, J. R. Cheeseman, G. Scalmani, V. Barone, G. A. Petersson, H. Nakatsuji, X. Li, M. Caricato, A. V. Marenich, J. Bloino, B. G. Janesko, R. Gomperts, B. Mennucci, H. P. Hratchian, J. V. Ortiz, A. F. Izmaylov, J. L. Sonnenberg, Williams, F. Ding, F. Lipparini, F. Egidi, J. Goings, B. Peng, A. Petrone, T. Henderson, D. Ranasinghe, V. G. Zakrzewski, J. Gao, N. Rega, G. Zheng, W. Liang, M. Hada, M. Ehara, K. Toyota, R. Fukuda, J. Hasegawa, M. Ishida, T. Nakajima, Y. Honda, O. Kitao, H. Nakai, T. Vreven, K. Throssell, J. A. Montgomery Jr., J. E. Peralta, F. Ogliaro, M. J. Bearpark, J. J. Heyd, E. N. Brothers, K. N. Kudin, V. N. Staroverov, T. A. Keith, R. Kobayashi, J. Normand, K. Raghavachari, A. P. Rendell, J. C. Burant, S. S. Iyengar, J. Tomasi, M. Cossi, J. M. Millam, M. Klene, C. Adamo, R. Cammi, J. W. Ochterski, R. L. Martin, K. Morokuma, O. Farkas, J. B. Foresman, D. J. Fox, Gaussian, Inc., Wallingford, CT, **2016**.

[11] M. K. Assefa, J. L. Devera, A. D. Brathwaite, J. D. Mosley, M. A. Duncan, *Chem. Phys. Lett.* **2015**, *640*, 175-179.
[12] S. Grimme, A. Hansen, S. Ehlert, J.-M. Mewes, *J. Chem. Phys.* **2021**, *154*, 064103.
[13] F. Neese, *WIREs Comput Mol Sci*. **2022**, *12*, e1606.
[14] A. D. Becke, *J. Chem. Phys.* **1993**, *98*, 5648-5652.
[15] R. Bjornsson, F. Neese, S. DeBeer, *Inorg. Chem.* **2017**, *56*, 1470-1477.
[16] A. Wolf, M. Reiher, B. A. Hess, *J. Chem. Phys.* **2002**, *117*, 9215-9226.
[17] a) S. Sinnecker, L. D. Slep, E. Bill, F. Neese, *Inorg. Chem.* **2005**, *44*, 2245-2254; b) F. Neese, *Inorg. Chim. Acta* **2002**, *337*, 181-192.
[18] a) A. D. Becke, *Phys. Rev. A* **1988**, *38*, 3098-3100; b) J. P. Perdew, *Phys. Rev. B* **1986**, *33*, 8822-8824; c) J. P. Perdew, *Phys. Rev. B* **1986**, *34*, 7406-7406; d) E. Caldeweyher, S. Ehlert, A. Hansen, H. Neugebauer, S. Spicher, C. Bannwarth, S. Grimme, *J. Chem. Phys.* **2019**, *150*.
[19] a) G. te Velde, F. M. Bickelhaupt, E. J. Baerends, C. Fonseca Guerra, S. J. A. van Gisbergen, J. G. Snijders, T. Ziegler, *J. Comput. Chem.* **2001**, *22*, 931-967; b) S. AMS 2023.1, Theoretical Chemistry, Vrije Universiteit, Amsterdam, The Netherlands, http://www.scm.com.
